# Commonly Reported Problems and Coping Strategies During the COVID-19 Crisis: A Survey of Graduate and Professional Students

**DOI:** 10.3389/fpsyg.2021.598557

**Published:** 2021-02-25

**Authors:** Akash R. Wasil, Rose E. Franzen, Sarah Gillespie, Joshua S. Steinberg, Tanvi Malhotra, Robert J. DeRubeis

**Affiliations:** ^1^Department of Psychology, University of Pennsylvania, Philadelphia, PA, United States; ^2^Children’s Hospital of Philadelphia, Philadelphia, PA, United States; ^3^Institute of Child Development, University of Minnesota, Minneapolis, MN, United States; ^4^Department of Psychology, Ashoka University, Sonipat, India

**Keywords:** coping strategies, top problems, evidence-based practices, public health, COVID-19

## Abstract

**Background:**

The COVID-19 crisis has introduced a variety of stressors, while simultaneously decreasing the availability of strategies to cope with stress. In this context, it could be useful to understand issues that people find most concerning and ways in which they cope with stress. In this study, we explored these questions with a sample of graduate and professional students.

**Method:**

Using open-ended assessments, we asked participants (*n* = 305) to identify their biggest challenge or concern (“top problem”), their most effective way of handling stress (“effective strategy”), and their most common way of handling stress (“common strategy”). We applied thematic analysis and evaluated whether participants’ strategies corresponded with evidence-based practices (EBPs).

**Results:**

Participants frequently reported top problems relating to productivity (27% of sample), physical health (26%), and emotional health (14%). Distraction was the most frequently classified common strategy (43%), whereas behavioral activation was the most frequently identified effective strategy (50%). Participants who reported a common strategy classified as an EBP reported lower depressive and anxiety symptoms. In contrast, there was no evidence of an association between symptom levels and whether or not participants’ effective strategy was an EBP. Participants who reported the same strategy as both their common and effective strategy (29%) reported lower depressive symptoms than those whose common and effective strategies were different.

**Conclusion:**

Our findings highlight stressors that students are experiencing and ways they are coping during the COVID-19 crisis. We discuss how these findings can inform mental health promotion efforts and future research on coping with stressors.

## Introduction

Coronavirus disease 2019 (COVID-19) has had an enormous public health impact. In addition to its serious physical health consequences, the virus and the resulting societal changes have had major impacts on the mental health of society (Li et al., 2020). The virus has introduced new stressors (e.g., fears of contracting the virus, concern for loved ones contracting the virus, economic uncertainty, job loss, social distancing) and challenges (e.g., maintaining strong social relationships while social distancing, staying productive while working from home).

We thought it would be useful to assess how people are *responding* to problems they are experiencing in the context of the COVID-19 pandemic and which problems they consider most important. Graduate students may find it more difficult to cope with existing problems in the context of new restrictions, or the pandemic may have introduced entirely new problems into their lives. In our view, understanding how individuals are responding to problems in their lives could be important for several reasons. First, individuals who are seeking advice (e.g., about how to navigate stressors relating to the pandemic) may be interested in learning about the coping strategies that others have found most effective (i.e., those that people have perceived as most helpful in coping with stress). Such strategies could also be included in outreach activities and could inform efforts to provide mental health advice to the public (Li et al., 2020). Second, mental health experts could prioritize evaluations of strategies that are *commonly used*, and those that are consistent with evidence-based practices (EBPs) could be promoted. Third, mental health professionals, policymakers, and public health officials could benefit from understanding the specific problems, stressors, and challenges that people perceive as most important during times of immense stress, such as this crisis. Research activities and funding targeted at problems that are commonly reported could be especially useful in combating the current crisis. Fourth, even beyond the COVID-19 crisis, such research could help us better understand adaptive ways of responding to stressful circumstances. Even under normal circumstances, researchers have been highly interested in emotion regulation (e.g., Aldao et al., 2010), coping with stressors (e.g., Littleton et al., 2007), and resilience in response to difficult circumstances (e.g., Hu et al., 2015). While the COVID-19 crisis represents a unique period in human history, some of the insights acquired during the COVID-19 crisis may generalize to other kinds of stressful situations. In summary, an assessment of top problems (i.e., the problems that people perceive as most stressful) and coping strategies (i.e., the strategies people are engaging in to handle stress) could have practical implications during the COVID-19 crisis while also generating knowledge that extends beyond the pandemic.

### Open-Ended Assessment

Although there are many measures of coping styles and common psychological problems, open-ended measures may be especially valuable. Many standardized measures of coping ask participants to respond to a set of predetermined items with predefined response options. In contrast, open-ended measures allow participants to freely report on their experiences without restriction. Closed-ended questionnaires have several strengths, including quantitative interpretations of scores, norms and benchmarks for comparison across different samples, and often well-documented psychometric integrity (Meyer et al., 2001). However, such measures also have a variety of important limitations. Closed-ended questionnaires of coping strategies limit the potential range of responses, decreasing our ability to thoroughly characterize and describe the strategies that people naturally use (Wasil et al., 2021). Furthermore, in the context of stressful situations like the COVID pandemic, many people may be employing coping strategies that are not well-captured on existing questionnaires. As a result, some standardized questionnaires may systematically miss coping strategies or problems that are unique to this specific period. Furthermore, closed-ended assessments of coping may include items that are no longer possible due to federal and local stay-at-home orders. For these reasons, open-ended measures may be useful in describing and characterizing peoples’ experiences during the COVID-19 crisis. Open-ended idiographic measures may be able to overcome some of these limitations and usefully complement closed-ended assessments. These measures allow participants to freely report on their experiences in an effort to maximize the relevance of the measure to each individual. Such measures may be especially valuable during the COVID crisis due to their flexibility (for a longer discussion of idiographic assessment see Haynes et al., 2009). Because an open-ended measure of coping would allow participants to list any kind of strategy, such a measure would allow policymakers and public health officials to understand the broad range of responses to emergencies. Furthermore, it is likely that the practicing social distancing has changed the types of coping strategies available to individuals (e.g., many individuals may not be able to go to the gym or seek in-person social support). Additionally, increased stress from the crisis may inhibit self-control (Duckworth et al., 2013), reducing peoples’ ability to select and execute appropriate coping strategies. Due to the novel context, an open-ended qualitative measure could be an important first step toward understanding coping responses and problems during the pandemic. After administering open-ended questionnaires, researchers could identify themes that are commonly reported. By first using idiographic assessments to understand the problems and concerns of people during this pandemic, researchers may be able to prioritize research questions and interventions that are most relevant to this pandemic.

Taken together, this logic suggests that the information acquired from open-ended measures could be especially useful for researchers, policymakers, public health officials who are trying to understand responses to stressful situations. Therefore, we employed open-ended questions prompting participants to identify, without restriction, the coping strategies that they perceive as most useful (i.e., “effective strategies”), coping strategies that they engage in most frequently (i.e., “common strategies”), and problems that they consider most important (i.e., “top problems”).

### Evidence-Based Practices and Coping Strategies

We also wanted to examine the extent to which peoples’ coping strategies mirrored treatment components in evidence-based psychotherapies. For several decades, scholars have tested mental health interventions, often in the form of published treatment manuals. Some scholars have identified evidence-based practices and principles (EBPs) that are commonly included within the treatment manuals of empirically supported treatments (Chorpita and Daleiden, 2009). For example, cognitive restructuring, behavioral activation, and problem solving are EBPs that are commonly found in interventions for depression (Chorpita and Daleiden, 2009). Some EBPs are thought to be active ingredients of change and have formed the basis of modular interventions (e.g., Weisz et al., 2012; Murray et al., 2014). More recently, scholars have been interested in examining the extent to which people *naturally* employ EBPs as coping strategies. In one study, middle school students with greater depressive symptoms were less likely to employ EBPs as coping techniques than students with fewer depressive symptoms (Ng et al., 2016).

These authors also distinguished between habitual responses (i.e., coping strategies that participants often employ) and perceived-effective responses (i.e., coping strategies that participants perceived as helping them feel better). We reasoned that a similar approach could be helpful in understanding coping strategies during the COVID-19 crisis. Specifically, we were interested in understanding an individual’s most common response to stress (hereafter referred to as an individual’s “common strategy”), the response that they perceived as his or her most effective (hereafter referred to as an individual’s “effective strategy”), and whether or not these strategies match. In a previous study, Ng et al. (2016) found that participants whose perceived-effective responses were the same as their habitual responses (referred to here as “matchers”) reported fewer depressive symptoms than those who reported different strategies (“non-matchers”). Furthermore, the regulatory fit framework proposes that coping strategies are most effective at regulating a stress response when individuals employ the strategies that they perceive as optimal (Bendezú et al., 2019). Thus, guided by prior empirical and theoretical work, we predicted that matchers would report lower symptomatology than non-matchers. We also reasoned that this would be true regardless of whether or not the strategy matchers perceived as most effective and most common could be classified as an EBP.

We also wondered if individuals employing EBPs as coping strategies during the COVID-19 crisis may be experiencing better mental health outcomes. A diathesis-stress framing suggests that, in non-stressful environments, individuals with and without effective coping strategies may experience similar psychological outcomes (Ingram and Luxton, 2005). However, in stressful environments, having effective coping skills to manage these stressors may protect against psychological distress. Indeed, coping responses are thought to be especially important protective factors during times of widespread community stress, including during epidemics, natural disasters, and wars (Xu and He, 2012; Rabelo et al., 2016; James et al., 2019).

Because EBPs are commonly included within the treatment manuals of empirically supported treatments and are thought to be active ingredients of change and efficacious means of managing mental health concerns (Chorpita and Daleiden, 2009), we predicted that individuals who listed an effective coping strategy that could be classified as an EBP would experience fewer depressive and anxiety symptoms than those who listed an effective coping strategy that could not be classified as an EBP. We reasoned that these individuals are aware of EBPs and find them personally useful for reducing stress, making them more likely to employ them than individuals who cannot identify an EBP as an effective strategy. Similarly, we predicted that individuals who listed a common coping strategy that could be classified as an EBP would experience fewer depressive and anxiety symptoms than those who listed a common coping strategy that could not be classified as an EBP. Because we hypothesized that more frequent implementation (i.e., more common utilization) of strategies that could be classified as EBPs would be associated with better outcomes, we also reasoned that the relationship between EBP endorsement (i.e., listing a coping strategy that could be classified as an EBP) and mental health outcomes would be stronger for common strategies than for effective strategies. In both cases, we reasoned that individuals who listed EBPs as coping strategies may be more likely to use these strategies in their everyday lives, and we reasoned that implementing EBPs as coping strategies may be associated with mental health outcomes (Ng et al., 2016). Because we hypothesized that implementing EBPs would be associated with better outcomes, we also reasoned that the relationship between EBP endorsement and mental health outcomes would be stronger for common strategies than for effective strategies.

### The Present Study

In this study, we administered open-ended questions to assess coping strategies and top problems among *n* = 305 graduate and professional students (referred to herein as “students” or as “participants”). Even before the COVID-19 pandemic, graduate students were vulnerable to a variety of mental health concerns including depression, anxiety, loneliness, and suicidal ideation (Evans et al., 2018). The COVID-19 crisis has exacerbated these concerns: many universities have ceased non-essential operations, mandated that students leave campus, and shut down university counseling centers. Thus, we were interested in examining the problems and coping strategies of students as they experienced the pandemic.

Our study has three aims. Our first aim (Aim 1) was to identify the frequencies of each effective strategy, common strategy, and top problem we identified. To that end, we analyzed the open-ended responses to identify commonly reported strategies and problems. Our second aim (Aim 2) was to identify potentially helpful coping strategies by examining associations among coping strategy use and mental health. We had three hypotheses. First, we hypothesized that those who identified EBPs as effective strategies would experience lower depressive symptoms and anxiety symptoms (Aim 2, Hypothesis 1). Second, we hypothesized the same trend for individuals who identified EBPs as common strategies (Aim 2, Hypothesis 2). Third, we hypothesized that matchers (individuals who report that their most common strategy is the same as their most effective strategy) will experience lower depressive symptoms and anxiety symptoms compared to non-matchers (Aim 2, Hypothesis 3). Our third aim was to test whether particular strategies or top problems were associated with higher symptoms (Aim 3). We discuss the implications of these findings for psychologists, higher education leaders, public health officials, and members of the general public.

## Materials and Methods

### Recruitment

The present study uses baseline data that were collected as part of an effort to disseminate a mental health promotion program to support graduate and professional students during COVID-19 (for additional details, see Wasil et al., 2020c). The project was conducted via a partnership with university deans and the Behavior Change for Good Initiative. On March 30 and March 31, 2020, an email message was sent out to a listserv of the university’s graduate and professional students. The email explained that we were launching an online single-session program grounded in behavioral science and designed to help students during the crisis. The email also included a link to the survey, hosted on Qualtrics. In the present study, we analyze responses from the first week of recruitment (i.e., March 30 to April 6).

### Procedure

Upon opening the Qualtrics link, participants were directed to a brief introductory screen with information about the study’s purpose and a general description of the activities. Participants then filled out a baseline questionnaire with measures of depressive symptoms, anxiety symptoms, secondary control, perceived ability to handle the COVID-19 crisis (described in further detail below). The questionnaire also included three open-ended questions asking participants to list their most effective coping strategy, most common coping strategy, and biggest problem. The present study uses information from the baseline questionnaire; details about the intervention are presented elsewhere (Wasil et al., 2020c). Study procedures were reviewed and deemed quality improvement by the University of Pennsylvania IRB.

### Measures

#### Depressive Symptoms (Patient Health Questionnaire-2)

The Patient Health Questionnaire-2 (PHQ-2; Kroenke et al., 2003), a commonly used measure of depression, was administered to participants at baseline. The PHQ-2 asks participants to report the frequency of depressed mood and anhedonia over the past 2 weeks. Each item is scored from 0 (“not at all”) to 3 (“nearly every day”). The PHQ-2 has demonstrated strong psychometric properties, including construct validity. PHQ-2 scores are associated with functional impairment, symptom-related difficulties, and clinician ratings of depression (Kroenke et al., 2003). Cronbach’s alpha in our sample was 0.8.

#### Anxiety Symptoms (Generalized Anxiety Disorder-2)

The Generalized Anxiety Disorder 2-item scale (GAD-2; Kroenke et al., 2007), a commonly used measure of anxiety, was administered to participants at baseline. The GAD-2 asks participants to report the frequency of anxiety and inability to stop worrying over the past 2 weeks. Each item is scored from 0 (“not at all”) to 3 (“nearly every day”). The GAD-2 has demonstrated strong psychometric properties, including construct validity. GAD-2 scores are associated with functional impairment, and clinician ratings of anxiety (Plummer et al., 2016). Cronbach’s alpha in our sample was 0.86.

#### Effective and Common Coping Strategies

Informed by idiographic approaches to measurement (Haynes et al., 2009), we asked participants to freely list their most effective and most common coping strategy. Participants received the following instructions:

We want to understand how you deal with negative emotions or stress. Please list your most **effective** strategy and most **common** strategy for trying to feel better when you’re feeling upset or stressed. Your most effective strategy might also be your most common strategy, or they might be different.

Then, participants received a write-in text box to list their most effective strategy and a separate box to list their most common strategy. This order was deliberate, so that the participants would report general coping strategies, rather than those that may be specific to the top problem they described.

#### Top Problem

Informed by previous research on open-ended assessments of problems (Weisz et al., 2011), we asked participants to list their biggest problem or concern. Participants received the following instructions:

We want to understand problems that are causing you stress or discomfort. Please list your biggest problem or concern below. Try to be as specific as possible.

Then, participants received a write-in text box to list their biggest problem or concern.

### Development of Coping Strategy Codebook

Our codebook of coping strategies was guided by our two main goals: (a) To examine the frequency of EBPs and (b) To identify commonly reported non-EBPs.

#### Selection of EBP Codes

We developed a list of EBPs by drawing from several sources. First, we reviewed a previous study which had applied a coding scheme of EBPs to coping strategies identified by middle school students (Ng et al., 2016). To supplement this existing taxonomy of EBPs, we reviewed treatment manuals for cognitive therapy, behavior therapy, and interpersonal therapy; each of which has been shown to be effective treatments for depression in children and adolescents (David-Ferdon and Kaslow, 2008). We also surveyed studies that have identified EBPs in youth psychotherapy manuals for depression (Chorpita and Daleiden, 2009) and anxiety (Higa-McMillan et al., 2016). Because these sources focused on common EBPs in *youth* psychotherapies, we also surveyed empirically supported treatment manuals for adults with depression and anxiety. This full search, distillation, and matching procedure is described elsewhere (for full details see Wasil et al., 2019, 2020a). In brief, we reviewed meta-analyses (e.g., Chambless and Hollon, 1998; Cuijpers et al., 2013) and relevant chapters of *A Guide to Treatments that Work* (Nathan and Gorman, 2015) to identify empirically supported interventions for adults. Then, we reviewed treatment manuals of empirically supported interventions (e.g., Barlow et al., 2010; Weissman et al., 2017) to identify EBPs. Finally, we reviewed literature on single-component “wise” interventions (Walton, 2014) and positive psychology interventions (Seligman et al., 2005; Bolier et al., 2013). These bodies of literature were important supplements to the psychotherapy elements given that our participants were not a treatment-seeking population.

One code, distraction, could not be neatly conceptualized as an EBP or as a non-EBP. For our distraction code, we used the definition applied by Ng et al. (2016). Although Ng et al. (2016) categorized distraction as an EBP, distraction is highly heterogeneous, and other scholars have conceptualized distraction as maladaptive or dysfunctional (e.g., Machado et al., 2020). Therefore, we perform one set of analyses with distraction as an EBP and one set with distraction as a non-EBP. We also describe the specific types of distraction that people reported and compare the kinds of distraction that people considered effective and those that they commonly employed.

#### Selection of Non-EBP Codes

Next, we identified coping strategies that were commonly reported but did not match EBPs. To identify these codes, we applied thematic analysis guidelines (Braun and Clarke, 2006). First, we familiarized ourselves with the data. The first author (*initials masked for review*), second author (*initials masked for review*), and fourth author (*initials masked for review*) independently reviewed effective coping responses and common coping responses. Then, they had open discussions to identify patterns and themes in the data. Through this process, an initial codebook was created to characterize themes that were frequently reported. Next, the first, second and fourth authors reviewed the datasets once more to identify additional themes that were not covered in the initial codebook drafts. Then, these three authors discussed their notes and produced a final version of the codebook. To assess inter-rater reliability, the second author and fourth author independently applied each codebook to 70 randomly selected responses. Coding was blinded (coders were not aware of whether responses were reported as common strategies or effective strategies). Cohen’s kappa was calculated for codes with at least 3 responses (Cohen’s kappa ranged from *k* = 0.70 to *k* = 1.00). Responses that did not fit into any category were labeled “miscellaneous” (*n* = 8 common responses and *n* = 9 effective responses). Then, both authors applied the codebook to the remaining responses. Disagreements were resolved via consensus between the first, second, and fourth authors.

Our final codebook for effective and common strategies included 29 codes that match EBPs and 6 codes that do not (see our Supplementary Material for a list of codes and definitions). We also included 23 subcodes, which allowed us to analyze specific approaches subsumed within larger codes (e.g., the “behavioral activation” code included subcodes for “physical activity” and “social activity”).

### Development of Top Problem Codebook

To develop our codebook of top problems, we applied thematic analysis (Braun and Clarke, 2006). Our process was nearly identical to the process we described above for the development and application of our coping strategy codebook (Cohen’s Kappa ranged from *k* = 0.78 to *k* = 1.0). The only difference was that the process involved the first author (*initials masked for review*), third author (*initials masked for review*), and fifth author (*initials masked for review*), whereas the development and application of the coping codebook involved the first, second, and fourth authors.

Next, we coded all responses according to whether the problem was definitely related to the COVID-19 pandemic (e.g., “my family becoming ill”), likely related (e.g., “loss of jobs/income”), or unlikely to be directly related (e.g., “the stability of my romantic relationship”). The third and fifth author applied these codes and obtained high agreement (*k* = 0.97).

### Analyses

To address our first aim, we assessed the frequency of each coping strategy and each top problem. We were especially interested in identifying strategies that were frequently reported as effective though not common (and vice-versa). Because our data were paired (i.e., each participant provided both a common and effective strategy), we performed an omnibus McNemar-Bowker chi-squared test with strategies that were listed by at least 5% of participants (i.e., behavioral activation, distraction, social support, and “other,” a category which consisted of the remaining responses). Then, we performed follow-up 2 × 2 McNemar tests to compare pairs of strategies (e.g., comparing the proportion of participants who listed behavioral activation as effective and distraction as common to the proportion who listed distraction as effective and behavioral activation as common).

To address our second aim, we tested three hypotheses related to coping strategies. First, we tested whether participants who identified an EBP as their most effective strategy reported lower depressive and anxiety symptoms. Second, we tested whether participants who identified an EBP as their most common strategy report lower depressive and anxiety symptoms. Third, we tested whether participants who reported the same strategy as their most effective and their most common reported lower depressive symptoms and anxiety symptoms. To test each of these hypotheses, we performed one-tailed *t*-tests.

Finally, to address our third aim, we examined if specific strategies and specific problems were associated with depressive symptoms and anxiety symptoms. To reduce the number of tests performed, we only ran tests that were adequately powered to detect a between-group effect size of *d* = 0.30 or greater. We conducted a power analysis to identify the minimum number of people we would require in each cell to detect our effect size of interest. As a result, we limited our analyses to those in which at least 18% of our sample (*n* = 55) endorsed a given strategy or problem. For each strategy or problem reported by at least 55 people, we analyzed its association with depressive symptoms and anxiety symptoms.

Hypotheses were stated prior to data analysis. Analyses were performed in R, and our code is available as Supplementary Material.

## Results

### Sample Characteristics

From 3/30/20 to 4/6/20, our survey received 561 clicks. Our sample for this present study consists of 305 individuals who began the baseline questionnaire and provided a response to our open-ended question about top problems and coping strategies. Demographic characteristics were collected at the end of the entire survey, so demographic characteristics are only available for participants who completed the survey. Demographic characteristics for these participants are reported in Table 1.

**TABLE 1:** Sample demographics.

	M (SD) or N (%)
N	305 (100%)
PHQ-2	2.04 (1.69)
GAD-2	2.68 (1.87)
Age	31.04 (8.91)
**Race/Ethnicity**	
White	114 (66.67%)
Asian	41 (23.98%)
Hispanic/Latinx/Spanish Origin	12 (7.02%)
Black	11 (6.43%)
Middle Eastern or North African	3 (1.75%)
Other	2 (1.17%)
Missing	134^*a*^
**Sex**	
Female	127 (72.99%)
Male	42 (24.14%)
Other	2 (1.15%)
Prefer not to answer	3 (1.72%)
Missing	131^*a*^
**Sexual orientation**	
Heterosexual or straight	140 (81.40%)
Bisexual	16 (9.30%)
Queer	10 (5.81%)
Fluid	6 (3.49%)
Gay or lesbian	5 (2.91%)
Pansexual	5 (2.91%)
Asexual	4 (2.33%)
Demisexual	3 (1.74%)
Questioning	3 (1.74%)
Prefer not to answer	5 (2.91%)
Missing	133^*a*^
**Social class (self-reported)**	
Poor	5 (2.89%)
Working class	27 (15.61%)
Middle class	111 (64.16%)
Affluent	30 (17.34%)
Missing	132^*a*^
**Experienced a mental illness (self-reported)**	
Yes	72 (41.62%)
Unsure	22 (12.72%)
No	79 (45.67%)
Missing	132^*a*^

**^*a*^Demographic data were collected after participants completed a 30-min online intervention. Missing data belong primarily to participants who filled out the baseline measures but did not complete the intervention.**

### Aim 1: Frequency of Top Problems and Coping Strategies

#### Top Problems

Table 2 presents the frequency of participants’ biggest problems. Productivity and work-related stressors (27.3%), Health concerns (25.6%), and Emotional Problems (13.8%) were the most frequently reported top problems. The majority of problems were coded as definitely related to COVID-19 (55.4%) or likely related (26.2%).

**TABLE 2:** Top problems reported during the COVID-19 pandemic.

Top problem	Percentage of people endorsing the problem (%)
Productivity/Work	27
Academic problems	15
Loss of productivity	10
Health	26
Loved ones	16
Personal health	10
World health	2
Frontline workers	1
Emotional problems	14
General uncertainty/anxiety	9
Existential crisis	2
Mental illness	1
Lack of control	1
Economic problems	13
Job	6
Economy	2
Social distancing/Travel restrictions	12
Loss of daily routine	5
Isolation/Loneliness	3
Far from home	1
Changes to plans/Goals	8
Miscellaneous	3
Altruism	3
Relationship problems	1
Other relationships	1
Roommates	0
News	0
No problems	0

#### Effective and Common Coping Strategies

Table 3 presents the frequency of coping strategies that participants found most effective (effective strategies) and used most commonly (common strategies). Table 3 includes the strategies that were endorsed by at least 5% of our sample (see our Supplementary Material for the full list of strategies and their frequencies). Behavioral Activation (49.1%), Distraction (16.1%), and Social Support (13.2%) were the most frequently reported effective strategies. Distraction (44.9%), Behavioral Activation (26.4%), and Social Support (9.2%) were the most frequently reported common strategies.

**TABLE 3:** Common and effective coping strategies reported during the COVID-19 pandemic.

Coping strategy	Percentage of people endorsing the strategy as a common strategy (%)	Percentage of people endorsing the strategy as an effective strategy (%)
**Distraction**	43	15
Behavioral distraction	42	14
TV	18	3
Food	9	1
Productivity	4	7
Social media	3	0
Reading	3	2
Music	2	0
Cognitive distraction	0	1
**Behavioral activation**	27	50
Physical activity	19	40
Going outside	5	14
Social activities	3	4
Routine	0	2
**Social support**	9	12
Friend	5	9
Family member	3	4
Significant other	1	2
Help	0	1
Feelings	0	1
**Other**	24	26
Any EBP (distraction included)	85	89
Any EBP (distraction excluded)	42	74

**Parent codes are bolded. The category “other” consists of strategies that were endorsed by less than 5% of participants.**

An omnibus McNemar-Bowker chi-squared test suggested that some strategies were more likely to be listed as effective though not common, while others were more likely to be listed as common though not effective (*X*^2^ = 70.37, *p* < 0.001). The follow-up tests revealed that distraction was more likely to be listed as a common strategy than an effective strategy when contrasted with each of the three other categories [behavioral activation (*X*^2^ = 40.02, *p* < 0.001); social support (*X*^2^ = 11.84, *p* < 0.001); and “other” strategies (*X*^2^ = 11.77, *p* < 0.001)]. We did not find evidence for any differences between behavioral activation and social support, behavioral activation and other strategies, or social support and other strategies (*p*s > 0.05).

As mentioned, we also assessed whether or not participants’ responses matched EBPs in empirically supported interventions. Given that some scholars have conceptualized distraction as an EBP (e.g., Ng et al., 2016), whereas others have conceptualized distraction as maladaptive (e.g., Machado et al., 2020), we performed two sets of analyses: one in which distraction was considered an EBP and one in which it was not. If distraction is considered an EBP, 89% of participants listed an EBP as their most effective strategy and 85% listed an EBP as their most common strategy. If distraction is not considered as an EBP, 73% of participants listed an EBP as their most effective strategy and 41% of participants listed an EBP as their most common strategy.

### Aim 2: Hypothesized Associations Between Coping Strategies and Mental Health

We hypothesized that individuals who listed EBPs as effective strategies or common strategies (i.e., “EBP endorsers”) would report fewer depressive symptoms and fewer anxiety symptoms than individuals who did not list an EBP as their effective strategy or common strategy (i.e., “non-EBP endorsers”). For each test, we performed a sensitivity analysis removing “Distraction” from our EBP list.

Table 4 shows the results of *t*-tests comparing depressive symptoms and anxiety symptoms between EBP endorsers and non-EBP endorsers.

**TABLE 4:** Relationship between evidence-based practice endorsement and mental health.

	EBP endorsement		Non-EBP endorsement			*t*-test results		
Mental health symptoms	Mean	SD	Mean	SD	*p*-value	Mean difference	Effect size (*d*)	Confidence interval (CI for Cohen’s *d*)
**Common strategy**								
Depressive symptoms	1.88	1.55	2.91	2.17	***p* = 0.002**	**1.03**	**0.62**	[0.30, 0.94]
Anxiety symptoms	2.53	1.77	3.54	2.20	***p* = 0.002**	**1.01**	**0.55**	[0.23, 0.87]
Depressive symptoms (without distraction as EBP)	1.63	1.47	2.34	1.78	***p* < 0.001**	**0.72**	**0.43**	[0.20, 0.66]
Anxiety symptoms (without distraction as EBP)	2.37	1.69	2.92	1.97	***p* = 0.006**	**0.55**	**0.30**	[0.07, 0.53]
**Effective strategy**								
Depressive symptoms	2.00	1.65	2.30	2.01	*p* = 0.17	0.004	0.18	[−0.19, 0.54]
Anxiety symptoms	2.69	1.88	2.67	1.83	*p* = 0.52	0.02	−0.01	[−0.37, 0.35]
Depressive symptoms (without distraction as EBP)	1.96	1.66	2.26	1.76	*p* = 0.09	0.30	0.18	[−0.08, 0.43]
Anxiety symptoms (without distraction as EBP)	2.72	1.88	2.58	1.87	*p* = 0.72	0.14	−0.08	[−0.34, 0.18]

**Significant p-values are bolded. Depressive symptoms were measured by the PHQ-2 and anxiety symptoms were measured by the GAD-2.**

#### Hypothesis 1: Reporting EBPs as Effective Strategies

Contrary to our hypothesis, we did not find that individuals who listed an EBP as their most effective coping strategy reported fewer depressive symptoms [EBP endorsers: *M* = 2.00, *SD* = 1.65; non-EBP endorsers: *M* = 2.28, *SD* = 2.00; *t*(302) = 0.96, *p* = 0.17, *d* = 0.18] or anxiety symptoms [EBP endorsers: *M* = 2.68, *SD* = 1.88; non-EBP endorsers: *M* = 2.67, *SD* = 1.83; *t*(302) = −0.06, *p* = 0.52, *d* = −0.01] than those who did not report an EBP as their most effective strategy. The relationship remained non-significant when distraction was not operationalized as an EBP for both depressive symptoms [EBP endorsers: *M* = 1.96, *SD* = 1.67; non-EBP endorsers: *M* = 2.26, *SD* = 1.76; *t*(302) = 1.34, *p* = 0.09, *d* = 0.18] and anxiety symptoms [EBP endorsers: *M* = 2.72, *SD* = 1.88; non-EBP endorsers: *M* = 2.58, *SD* = 1.87; *t*(302) = −0.59, *p* = 0.82, *d* = −0.08]. Thus, our first hypothesis (that endorsement of an effective strategy that matched an EBP would be associated with lower depressive and anxiety symptoms) was not supported.

#### Hypothesis 2: Reporting EBPs as Common Strategies

Consistent with our second hypothesis, we found that individuals who listed an EBP as their most common coping strategy reported fewer depressive symptoms [EBP endorsers: *M* = 1.88, *SD* = 1.55; non-EBP endorsers: *M* = 2.91, *SD* = 2.17; *t*(53.49) = 3.08, *p* = 0.002, *d* = 0.62] and fewer anxiety symptoms [EBP endorsers: *M* = 2.53, *SD* = 1.77; non-EBP endorsers: *M* = 3.54, *SD* = 2.20; *t*(55.98) = 2.95, *p* = 0.002, *d* = 0.55] than those who did not report an EBP as their most common strategy. The effect remained significant when distraction was not operationalized as an EBP; our hypothesis was supported for reported depressive symptoms [EBP endorsers: *M* = 1.63, *SD* = 1.47; non-EBP endorsers: *M* = 2.34, *SD* = 1.78; *t*(296.34) = 3.83, *p* < 0.0001, *d* = 0.43] and anxiety symptoms [EBP endorsers: *M* = 2.37, *SD* = 1.69; non-EBP endorsers: *M* = 2.92, *SD* = 1.97; *t*(301) = 2.56, *p* = 0.006, *d* = 0.30]. Thus, our second hypothesis (that endorsement of a common strategy that matched an EBP would be associated with lower depressive and anxiety symptoms) was supported.

#### Hypothesis 3: Match Between Common Strategy and Effective Strategy

We hypothesized that individuals who listed their most effective strategy as their most common strategy (i.e., “matchers”) would report fewer depressive symptoms and anxiety symptoms than those who did not list the same strategy for both questions (i.e., “non-matchers”). In our sample, 29% of participants were matchers and 71% were non-matchers. Consistent with our hypothesis, individuals whose common strategy matched their effective strategy reported fewer depressive symptoms [matchers: *M* = 1.64, *SD* = 1.46; non-matchers: *M* = 2.20, *SD* = 1.76; *t*(301) = 2.61, *p* = 0.005, *d* = 0.33]. This trend was not statistically significant for anxiety symptoms [matchers: *M* = 2.53, *SD* = 1.73; non-matchers: *M* = 2.75, *SD* = 1.93; *t*(301) = 0.93, *p* = 0.18, *d* = 0.12]. Thus, our third hypothesis was partially supported.

### Aim 3: Exploratory Associations Between Specific Strategies, Top Problems, and Mental Health

#### Associations Between Coping Strategies and Mental Health

As exploratory analyses, we examined the relationship between specific strategies (with at least 18% endorsement as either common or effective) and mental health problems (Table 5).

**TABLE 5:** Relationship between coping strategies and mental health during COVID-19.

	Common strategy					Effective strategy				
	Strategy endorsed		Strategy not endorsed			Strategy endorsed		Strategy not endorsed		
	Mean	SD	Mean	SD	Effect size (*d*) and CI	Mean	SD	Mean	SD	Effect size (*d*) and CI
**Behavioral activation**										
Depression	1.59	1.52	2.20	1.72	**0.37 [0.11, 0.62]**	2.09	1.72	1.98	1.66	−0.07 [−0.29, 0.16]
Anxiety	2.31	1.70	2.82	1.92	**0.27 [0.02, 0.53]**	2.81	1.92	2.56	1.81	−0.14 [−0.36, 0.09]
Distraction										
Depression	2.11	1.58	1.98	1.77	−0.08 [−0.31, 0.15]	2.22	1.58	2.00	1.71	−0.13 [−0.45, 0.19]
Anxiety	2.68	1.84	2.68	1.90	0.002 [−0.23, 0.23]	2.51	1.91	2.71	1.87	0.107 [−0.21, 0.42]
**Physical activity**										
Depression	1.19	1.19	2.23	1.73	**0.63 [0.34, 0.92]**	2.08	1.64	2.01	1.73	−0.05 [−0.28, 0.18]
Anxiety	2.09	1.71	2.82	1.88	**0.39 [0.10, 0.69]**	2.88	1.96	2.55	1.80	−0.17 [−0.40, 0.06]
**Television**										
Depression	2.11	1.57	2.02	1.72	−0.05 [−0.35, 0.24]	2.10	2.28	2.03	1.67	−0.04 [−0.67, 0.59]
Anxiety	2.58	1.72	2.70	1.91	0.07 [−0.23, 0.36]	2.60	2.17	2.68	1.86	0.05 [−0.59, 0.68]

**Significant p-values are bolded. Depressive symptoms were measured by the PHQ-2 and anxiety symptoms were measured by the GAD-2.**

Individuals who reported behavioral activation (BA) as their common coping strategy reported fewer depressive symptoms [BA-endorsers: *M* = 1.59, *SD* = 1.52; non-BA endorsers: *M* = 2.20, *SD* = 1.72; *t*(303) = 2.85, *p* = 0.005, *d* = 0.37] and anxiety symptoms [BA-endorsers: *M* = 2.31, *SD* = 1.70; non-BA endorsers: *M* = 2.82, *SD* = 1.92; *t*(303) = 2.12, *p* = 0.035, *d* = 0.27] than those who did not. A stronger effect was found when comparing individuals who endorsed physical activity as a common strategy to those who did not. Individuals who reported physical activity (PA) as their common coping strategy reporter fewer depressive symptoms [PA-endorsers: *M* = 1.19, *SD* = 1.19; non-PA endorsers: *M* = 2.23, *SD* = 1.73; *t*(117.57) = 5.41, *p* < 0.000001, *d* = 0.63] and anxiety symptoms [PA-endorsers: *M* = 2.09, *SD* = 1.71; non-PA endorsers: *M* = 2.82, *SD* = 1.88; *t*(303) = 2.69, *p* = 0.008, *d* = 0.39] than those who did not.

We did not find a statistically significant difference in depressive symptoms or anxiety symptoms based on whether participants endorsed distraction as a common strategy (*p*s > 0.48) or as an effective strategy (*p*s > 0.42).

#### Associations Between Top Problems and Mental Health Outcomes

As exploratory analyses, we examined the relationship between specific top problems (with at least 18% endorsement) and mental health problems. We did not find a statistically significant difference in depressive symptoms or anxiety symptoms based on top problem endorsement (*p*s > 0.10).

## Discussion

We administered open-ended assessments to survey graduate and professional students about the top problems they are encountering during the COVID-19 pandemic and the coping strategies they find effective and use commonly. The majority of problems (81.6%) were coded as explicitly related to COVID-19 or likely related, due to widespread changes to daily life in response to the virus. We found that most participants were concerned about problems related to productivity and work-related stressors, health concerns, and emotional problems in this new context. Furthermore, many of the coping strategies that participants reported as being their most effective or most common strategy frequently corresponded with components of evidence-based interventions. We hypothesized that reporting an EBP as an *effective strategy* (hypothesis 1) or as a *common strategy* (hypothesis 2) would be associated with lower depressive and anxiety symptoms. However, we found that only those who reported an EBP as a common strategy endorsed significantly lower symptoms. Additionally, we hypothesized that individuals whose common strategy and effective strategy were the same would experience fewer depressive symptoms and anxiety symptoms (hypothesis 3). We found that individuals who commonly employ their most effective strategies (i.e., “matchers”) had lower depressive but not anxiety symptoms, providing partial support for that hypothesis.

Behavioral activation was the most frequently reported effective strategy, whereas distraction was the most frequently reported common strategy. Behavioral activation is a core component of many empirically supported interventions for depression, and treatments targeting engagement in enjoyable activities and reward sensitivity through behavioral activation are effective for depression and anxiety (Dimidjian et al., 2006; Craske et al., 2019). Additionally, randomized trials and meta-analyses support the efficacy of behavioral activation as a treatment for depression (Cuijpers et al., 2007; Gawrysiak et al., 2009; Dimidjian et al., 2011; Patel et al., 2017). Importantly, our study offers a unique finding about behavioral activation: people appear to use it commonly and to perceive it as effective, and those who use it commonly report fewer internalizing symptoms. In contrast, few participants reported using most of the other elements of empirically supported interventions (e.g., cognitive restructuring, problem solving, exposure). This suggests that behavioral activation, compared to other EBPs, is relatively commonly used to reduce stress and improve well-being. If replicated, these findings would suggest that interventions centered on behavioral activation may often harness participants’ existing habits and techniques, whereas interventions centered on other components may involve teaching entirely new skills. Our findings also highlight some specific forms of behavioral activation that are especially common: most of the behavioral activation responses involved exercise or outdoor activities. One randomized controlled trial conducted with young adults demonstrated that a brief period of aerobic exercise improved emotion regulation following a negative mood induction (Bernstein and McNally, 2017). Additionally, physical activity is prospectively associated with lower risk of depression (for a review, see Firth et al., 2020). Participants who use exercise to cope with stress may benefit from both acute and long term effects of this form of behavioral activation.

Furthermore, even though half of our sample reported behavioral activation as their most effective strategy, only a quarter reported it as their most common strategy. This “common-effective gap” suggests that much of our sample may benefit from implementing behavioral activation strategies that they already view as effective. In contrast, about half of our sample reported distraction as their most common strategy, yet only 15% reported it as their most effective strategy. While behavioral activation might be underutilized, distraction might be overutilized in the context of the COVID-19 pandemic. Specific forms of distraction may be especially overutilized: watching television (18%) and eating (10%) were the most commonly reported kinds of distraction, yet very few participants listed these strategies as their most effective strategy (<1 and 3%, respectively). It is possible that behavioral activation is viewed as relatively effortful, while distraction is viewed as easier to implement (albeit less effective for addressing distress). Furthermore, certain kinds of behavioral activation (e.g., going outside, performing in-person activities with friends) may have been limited by measures designed to stop the spread of COVID-19, whereas certain kinds of distraction (e.g., watching TV, eating food) might have remained accessible. Among a cross-national survey of 551 adults, 73.7% self-reported increases in “binge watching” behavior as a result of the pandemic (Dixit et al., 2020). Future research is needed to understand why, and under which circumstances, individuals turn to certain coping strategies.

The heterogeneity in the types of distraction reported may contribute to the literature on whether this form of coping confers psychological benefits and for whom. In some therapy modalities, such as acceptance and commitment therapy, distraction is not always considered maladaptive (Blackledge and Hayes, 2001). Some forms of distraction can be considered helpful in moderation (e.g., drinking a glass of wine to unwind at night). However, when distraction becomes excessive, inflexible, or uncontrollable, it is more likely to be maladaptive (Harris, 2006). In our sample, the most frequently reported forms of distraction were watching television, eating food, and trying to stay productive. Distinguishing between these forms of distraction may be important, as some forms of distraction are generally more adaptive (e.g., listening to music) than others (e.g., substance use). Furthermore, it is likely that distraction is more effective in certain contexts and for certain individuals than others. For instance, the regulatory-fit framework asserts that the effectiveness of coping strategies may vary according to individual and contextual differences and that coping is maximally effective when an individual uses their own optimal strategy (Bendezú et al., 2019). Future research is needed to understand which types of distraction are effective, for whom they are most helpful, and under which circumstances they are most adaptive.

Finally, some types of distraction may be adaptive for some individuals during acute stress (Janson and Rohleder, 2017), such as the onset of the pandemic when mental health symptoms peaked before declining (Daly et al., 2020), whereas a consistent pattern of passive coping strategies may be maladaptive in the long term (Fledderus et al., 2010). Over four waves of data collection at various points in the pandemic, Bendau et al. (2020) found that suppression of pandemic-related thoughts and a decreased healthy diet were associated with worsening mental health, while higher acceptance of the situation was associated with improvements. Our data were collected in late March and April of 2020, when participants might have experienced acute, recent changes to daily life, accompanied by new fears and worries. As the COVID-19 crisis stretches on, longitudinal research may reveal whether different strategies are needed for promoting well-being during a more prolonged crisis.

Our findings suggest that *knowing* which strategies are effective for managing one’s own emotions may not improve mental health outcomes during times of stress; what appears to be helpful is *implementing* those strategies (i.e., commonly using strategies that one finds effective, like “matchers”). Although our findings are cross-sectional, it is noteworthy that listing an EBP as an effective strategy was not associated with mental health outcomes while listing an EBP as a common strategy was associated with lower depressive symptoms and anxiety symptoms. When asked to identify their most effective strategy, nearly all of our participants listed one that was coded as similar to strategies taught in empirically supported treatments. In contrast, relatively few individuals were commonly executing coping strategies that matched EBPs or that they themselves viewed as most effective (71% were non-matchers). We also found that those who commonly employed their most effective strategy (i.e., “matchers”) report less severe symptoms of depression. These findings echo recent discussions in the science of behavior change. Research on behavior change has shown that people commonly experience conflicts between what they “want” to do and what they know they “should” do in order to feel better (Milkman et al., 2008). In the context of the pandemic, people may face additional barriers to implementing the strategies that they themselves know they “should” do in order to feel better (i.e., their most effective strategy). This finding suggests that interventions could help individuals identify the strategies that they find useful, encourage them to engage in such techniques, and problem-solve around barriers to implementing them in the pandemic context (e.g., substitute similar activities that allow for social distancing). In some cases, however, an individual may not be able to access their most effective strategies (e.g., due to environmental or economic constraints) or individuals may perceive maladaptive strategies as effective (e.g., excessive use of drugs or alcohol). Thus, interventions that focus on helping people employ strategies they perceive as effective may not be helpful in every case. Future research could examine if, when, and for whom such interventions are appropriate.

Our findings also offer suggestions that can inform efforts to help people cope with stress during the pandemic. Importantly, individuals who are commonly employing EBPs reported better mental health. While not conclusive, this finding supports the idea that teaching people to use EBPs in daily life could prepare them to cope effectively in stressful situations; such skills may be particularly valuable for buffering risk in stressful environments. Additionally, it is notable that 13 of our 29 EBP codes were not reported by any participant as a common or as an effective strategy, including exposure, finding meaning, and self-monitoring. Others were mentioned rarely, such as reframing (1% listed as effective, 0% as common) and relaxation (4% as effective, 1% as common). Interestingly, cognitive coping strategies were extremely rare relative to behavioral strategies. This is especially surprising, given that reappraisal is a highly studied emotion regulation strategy and cognitive restructuring is a well-studied tool in several mental health interventions (Aldao et al., 2010).

Future research is needed to understand why these specific EBPs are uncommonly used. One possibility is that strategies like reframing and relaxation are often subsumed under other strategies in our codebook. For example, if a person listed “talking to a friend” as a coping strategy (coded as “social support”), we would not be able to identify if these conversations involved changing one’s beliefs, making meaning out of a difficult situation, distracting oneself from a problem, relaxing, or several other coping strategies. It is also possible that these strategies are not considered helpful in everyday coping or are more difficult to implement without guidance from a therapist or self-guided intervention. Finally, it is possible that these strategies would be helpful, but most people are not aware of them. In this case, disseminating information about these strategies and including them in interventions might be especially important. Additional research is needed to understand whether mental health professionals should prioritize teaching people new coping strategies or training people to use existing coping strategies in new ways. Such research could inform a related body of work, examining whether clinicians should focus on amplifying clients’ strengths or working on their weaknesses (Cheavens et al., 2012).

Our findings also suggest that behavioral activation, and especially physical activity, may be particularly important during the crisis. Although physical activity was one of the most frequently reported effective coping strategies, it was less frequently listed as a common strategy. During the pandemic, unfortunately, individuals’ options for physical activity have been limited. In order to safely practice social distancing, many gyms have closed and many individuals are limiting the time they spend outside; some participants even listed this as their top problem (e.g., “Haven’t been able to participate in my main stress reducing activities: gym and Brazilian Jiu Jitsu”). Such strategies, even if psychologically helpful and even necessary for minimizing viral transmission in the short-run, may lead to important health consequences in the long-run. For example, many of our participants reported common coping strategies that involve being sedentary (e.g., watching television, using social media, and refraining from activity) or consuming food or alcohol, trends which have been reported in other articles (Alomari et al., 2020; Dixit et al., 2020). Importantly, some cities have taken innovative approaches to making physical activity safe and accessible. For instance, Minneapolis and St. Paul closed roads around popular parks to allow pedestrians and cyclists room to maintain safe distances (Ojeda-Zapata, 2020). Future research could specifically ask participants about barriers to using one’s most effective strategies in order to identify policy approaches that balance physical and mental health considerations. Interventions that encourage the use of effective and health-promoting coping strategies may be important for both short-term mental health and long-term physical health (Bernstein and McNally, 2017). Additionally, efforts to practice behavioral activation and other EBPs in the context of the pandemic may be especially important. Several of these techniques are present in popular digital mental health interventions (Wasil et al.,2020a,b), which could be especially useful during the pandemic and future public health emergencies.

Our findings should be interpreted in light of several limitations. Notably, our findings focus specifically on graduate and professional students; future research is needed to understand if our findings replicate among other populations. Thus, although the mental health of students is essential during the crisis, these findings may not generalize to other groups—especially those who are more proximally affected by the crisis (e.g., healthcare workers). There are also important regional differences in how people are affected by the pandemic. Our sample comes from a university in an urban area of the Northeast and may not fully generalize to other regions. Additionally, the limited range of our depression and anxiety measures (scores on each measure range from 0 to 6), as well as limited variability in our sample, may have reduced our ability to detect effects. We also only administered questionnaires measuring the two most common mental health problems (depression and anxiety). Further research is needed to understand other mental health problems in the context of the pandemic. Future research may also help us understand how people apply coping strategies in response to specific kinds of stressors. It is possible that certain kinds of stressors are more likely to evoke certain kinds of coping strategies (e.g., pervasive stressors may elicit different kinds of coping strategies than acute stressors). Furthermore, our data are cross-sectional, meaning that our inferential statistics are not sufficient to draw causal claims. Finally, we did not restrict participants to list problems or coping strategies that were caused by the pandemic; participants were allowed to list problems and strategies that were present prior to the pandemic. This choice was intentional because we wanted to understand participants’ problems and strategies, regardless of whether or not these problems were caused by the pandemic or these strategies were employed as a result of the pandemic. Thus, future research is needed to understand which kinds of problems and which kinds of strategies are used in direct response to certain stressors.

While our study enabled us to identify coping strategies, future research could probe the quality, frequency, and promoters/limiters of these strategies. Though two individuals report activities that can be classified as behavioral activation, one may be doing so in a way that is more consistent with the ways it would be taught in an empirically supported treatment. Understanding the extent to which individuals are employing these strategies with high or low success could point to opportunities for refining the strategies that individuals are using. Additionally, in future research with the Top Problems Assessment, it could be useful to acquire more information about participants’ problems. Specifically, it may be useful to assess the severity of the problem, when the problem began, and how often the problem occurs. Such information could help researchers understand if participants respond differently to different kinds of problems (e.g., acute vs. chronic). Future research could also include follow-up studies that longitudinally track or experimentally manipulate the use of coping strategies. Intervention studies could be used to support participants in using the coping strategies that are perceived to be effective; this could simultaneously benefit participants during this crisis and test underlying theories about how coping strategies relate to distress beyond the scope of this pandemic.

## Data Availability Statement

The datasets for this article are not publicly available because the authors did not receive ethical clearance to share the data collected for this project. The R code associated with this project has been made available as Supplementary Material. Requests to access the datasets should be directed to AW, wasil@sas.upenn.edu.

## Ethics Statement

The studies involving human participants were reviewed and approved by the University of Pennsylvania Institutional Review Board. The ethics committee waived the requirement of written informed consent for participation.

## Author Contributions

AW conceptualized the idea for the study with RD. RF, SG, JS, and TM applied the codebooks. AW performed data analysis with oversight from RD and support from RF, SG, JS, TM, and RD. AW wrote the initial R script. RF, SG, JS, and TM reviewed the script. All authors contributed to the development of the codebooks, writing, and revising the manuscript.

## Conflict of Interest

The authors declare that the research was conducted in the absence of any commercial or financial relationships that could be construed as a potential conflict of interest. The reviewer JB declared a shared affiliation with one of the authors, SG, to the handling editor at time of review.
