# Supplementary material for: Commonly Reported Problems and Coping Strategies During the COVID-19 Crisis: A Survey of Graduate and Professional Students
Source: Front Psychol. 2021 Feb 25;12:598557. doi: 10.3389/fpsyg.2021.598557 (PMC7947789; doi:10.3389/fpsyg.2021.598557)
Supplement: Supplementary file 1 [file Table_1.DOCX]

**Supplementary File One: Codes and Definitions**

Table S1 shows a complete list of codes and definitions. We also include the source(s) from which the codes were taken. For some codes, we used definitions from previous studies; these codes are presented in quotations. Codes receiving a “(P)” are “parent” codes and represent a broad category, with codes receiving a “(S)” being more specific sub-codes of the preceding parent codes. Codes given “(SS)” are sub-sub-codes (sub-codes of the preceding sub-codes).

| **Code** | **Definition** | **Sample Response** |
| --- | --- | --- |
| **(P) Behavioral Activation**  (Ng, Eckshtain, & Weisz, 2016) | “Engaging in activities that are enjoyable, use  up energy, or involve friends/family, success, creativity...or helping others”. | “Doing an activity like making a craft or cooking a complicated recipe” |
| **(S) Physical Activity** | E.g., working out, walking, biking. | “Getting exercise” |
| **(S) Social Activities** | Activities that involve others (e.g., playing board games, making a bonfire with friends, etc.). Not to be given if the strategy involves seeking support from a loved one (see “Increasing Social Support”). | “Zoom call with friends” |
| **(S) Going Outside** | Leaving one’s home for the purpose of going outside. Not to be given if the activity involves leaving one’s house to go to another indoor location. | “Walking outside, gardening” |
| **(S) Routine** | Creating and/or sticking to a routine. | “Following a routine that includes work, sleep, exercise, gardening, pets, and social connection while allowing plenty of breathing room for emotions, leaning into uncertainty, and rest…” |
| **(P) Increasing Social Support**  (Ng et al., 2016) | “Talking with others, getting advice,  information, understanding, and comfort from others, acquiring/strengthening relationships”. | “Talk to people who fill me up and listen well and also have thoughtful approaches to their days” |
| **(S) Family Member** | Seeking support from a family member. | “Talking with...family” |
| **(S) Significant Other** | Seeking support from a significant other. | “Talking to my partner” |
| **(S) Friend** | Seeking support from a friend. | “Reaching out to friends/supports in my mutual aid fellowship.” |
| **(S) Help** | Requesting help from someone. | “talk to people who can help me or encourage me” |
| **(S) Feelings** | Expressing feelings to someone. | “Talking to others about how I feel” |
| **(P) Problem Solving**  (Ng et al., 2016) | “Identifying the problem, generating solutions and weighing their pros and cons, taking action, evaluating the outcome, anticipating or preventing future problems, and social problem solving including assertiveness, negotiation, empathy, communication analysis, active listening, warm or nonblaming responses”. | “Coming up with things I can do to resolve the stressor and acting on them” |
| **(P) Humor** | Making a joke/laughing at a joke or finding the humor or irony of a situation. | Not available because none of the responses received this code |
| **(P) Distraction**  (Ng et al., 2016) | “Thinking about something else, doing something to occupy oneself”. | “Distracting myself” |
| **(S) Cognitive**  (Ng et al., 2016) | “Thinking about something else...to occupy oneself”. | “Focus on something that will make me feel better or have a different thought” |
| **(S) Behavioral**  (Ng et al., 2016) | “Doing something to occupy oneself”. | “Changing activities” |
| **(SS) Productivity** | Working through tasks that need to get done but are not necessarily related to the problem (e.g., cleaning, paying bills, doing homework). | “Accomplishing the tasks on my to-do list” |
| **(SS) Music** | Listening to music. | “Taking time to listen to music I enjoy” |
| **(SS) Social Media** | Engaging with social media websites or applications. | “Going on social media” |
| **(SS) Reading** | Reading (non-schoolwork). | “Reading a book” |
| **(SS) TV** | Watching television, movies, or other videos. | “Watching trashy reality television” |
| **(SS) Food** | Eating food or drinking (non-alcoholic beverages only). | “Comfort eating” |
| **(P) Substance Use** | Consuming drugs and/or alcohol. | “Drinking wine” |
| **(P) Reframing**  (Referred to as “Cognitive Strategies” in Ng et al., 2016) | "Evaluating the accuracy or helpfulness of thoughts, generating realistic or positive alternatives, thinking about the good sides of a bad situation, thought interruption, setting aside worry time, identifying negative thoughts and their link to feelings and behavior”. | “Focusing on the positives, putting my situation into perspective” |
| **(P) Practice or Perseverance**  (Ng et al., 2016) | “Practicing to build skills or to do better at something, homework, role plays, keep trying, try again next time”. | Not available because none of the responses received this code. |
| **(P) Avoidance** | Avoiding the problem cognitively and/or behaviorally. Does not involve replacing the undesired cognitions or behaviors with different cognitions or behaviors (see “Distraction”). | “I try not to think about it” |
| **(P) Relaxation**  (Chorpita & Daleiden, 2009)  (Higa-McMillan, Francis,  Najarian, & Chorpita, 2016)  (Ng et al., 2016) | Techniques designed to relax the body, such as “deep breathing, progressive muscle relaxation, positive imagery”.  (Ng et al., 2016) | “Deep breathing” |
| **(P) Goal Setting**  (Ng et al., 2016) | “E.g., identifying goals and barriers to goals, breaking goals into smaller steps, planning to achieve goal, charting progress, evaluating outcomes”. | “Talk about it, reassess the situation, and set new goals” |
| **(P) Modeling**  (Ng et al., 2016) | “Using others as a guide for how to act”. | Not available because none of the responses received this code. |
| **(P) Journaling** | Keeping a journal or a diary. | “Journaling” |
| **(P) Self-Monitoring**  (Ng et al., 2016) | “Collecting information on one's feelings, behaviors and events”. | Not available because none of the responses received this code. |
| **(P) Psychoeducation**  (Chorpita & Daleiden, 2009)  (Higa-McMillan et al., 2016)  (Lindsey et al., 2014) | Seeking to understand information related to mental health or wellness. Can include research regarding coping strategies and the etiology of mental health concerns. | Not available because none of the responses received this code. |
| **(P) Reinforcement**  (Ng et al., 2016) | “Making a plan to reward self for working harder or doing better”. | Not available because none of the responses received this code. |
| **(P) Identifying Stress-Related Situations**  (Ng et al., 2016) | “Identifying situations that make one feel tense or uncomfortable”. | Not available because none of the responses received this code. |
| **(P) Understanding Affect**  (Ng et al., 2016) | “Labeling emotions, understanding situations leading to positive or negative feelings”. | “name and explore my feelings” |
| **(P) Understanding and Finding Meaning in Loss and Change**  (Ng et al., 2016) | “Mourning death or relationship loss, assessing losses and gains from change, identifying new demands”. | Not available because none of the responses received this code. |
| **(P) Personalizing Treatment**  (Ng et al., 2016) | “Identifying best-fit skills for oneself”. | Not available because none of the responses received this code. |
| **(P) Building a Positive Sense of Self**  (Ng et al., 2016) | “Recognizing strengths, broadening  self-definition”. | Not available because none of the responses received this code. |
| **(P) Understanding Relationships and their Link to Affect**  (Ng et al., 2016) | “Identifying important relationships, recognizing that they influence mood”. | Not available because none of the responses received this code. |
| **(P) Limited Sick Role**  (Ng et al., 2016) | “Acknowledging that when depressed, one cannot do things as well as before, but will gradually increase activities and responsibilities”. | Not available because none of the responses received this code. |
| **(P) Exposure**  (Chorpita & Daleiden, 2009)  (Higa-McMillan et al., 2016) | Exposure to a source of anxiety or discomfort with therapeutic intent, and without the intent to harm. Includes interoceptive exposures (exposures to physical feelings), imagined exposures (imagining scenarios that evoke discomfort), and in-vivo exposures (real-world contact with sources of discomfort). | Not available because none of the responses received this code. |
| **(P) Guided Imagery** | Visualizing images (e.g., beach, ocean) or situations (e.g., oneself giving a good presentation). | Not available because none of the responses received this code. |
| **(P) Religion** | E.g., praying, reading religious texts. | “pray and remember that God is in control of everything and has purpose even when I don't understand what's going on” |
| **(P) Mindfulness/Meditation**  (Strosahl & Robinson, 2017) | Activities that guide practice of focusing attention in order to achieve a calmer, clearer state of mind, or a meditation exercise | “mindfulness - becoming aware of negative thoughts and feelings, labeling them, and letting them go” |
| **(P) Values**  (Strosahl & Robinson, 2017) | Considering and identifying values, or the things that are most important to a person (e.g., kindness, family, learning). Usually also involves considering ways in which a person can better live up to their values. | Not available because none of the responses received this code. |
| **(P) Kindness to Self**  (Strosahl & Robinson, 2017) | Encouraging and guiding a person to be kind to themselves, often through considering good personal qualities and evidence for them, or by encouraging self-forgiveness or acceptance for perceived flaws and mistakes. | “Being kind and patient with myself” |
| **(P) Kindness to Others**  (Strosahl & Robinson, 2017) | Activities that involve showing kindness to another person. | Not available because none of the responses received this code. |
| **(P) Gratitude** | Expressing gratitude to someone or reflecting on things for which one can be grateful (e.g., gratitude journal, reflecting on good things that have happened in one’s life). | “Trying to think of what's good in life” |
| **(P) Refraining from Activity** | Withdrawing or not participating in activities. | “sitting and doing nothing” |
| **(S) Sleep** | Sleep, naps. | “Taking a nap” |
| **(P) Non-communicative Expression of Feelings** | E.g., crying, hitting, screaming. | “Crying” |
| **(S) Crying** | Crying. | “Crying” |
| **(S) Externalizing Behavior** | Lashing out, hitting, being destructive. | Not available because none of the responses received this code. |
| **(P) Information Seeking** | Searching for information related to the pandemic. | “Reading more about COVID-19 so I can understand the current situation” |
| **(P) Avoiding News** | Removing/ignoring news sources. | “Stop watching the news about coronavirus” |
| **(P) Nothing** | Indicating that one is not sure what they do that is common/effective or that they do not do anything in response to stress. | “not really doing anything, since when i am feeling down i don't have the interest in trying to help myself” |
| **(P) Miscellaneous** | Response not able to be categorized by the above codes. | Any code that does not fit into any of the above categories would receive this label. The response below is simply one example:.  “Letting myself "feel" that feeling for a few minutes, then moving on to something relaxing or more productive.” |

**References**

Chorpita, B., & Daleiden, E. L. (2009). Mapping evidence-based treatments for children and adolescents: Application of the distillation and matching model to 615 treatments from 322 randomized trials. Journal of Consulting and Clinical Psychology. <https://doi.org/10.1037/a0014565>

Higa-McMillan, C. K., Francis, S. E., Rith-Najarian, L., & Chorpita, B. F. (2016). Evidence Base Update: 50 Years of Research on Treatment for Child and Adolescent Anxiety. *Journal of Clinical Child & Adolescent Psychology*, *45*(2), 91–113.<https://doi.org/10.1080/15374416.2015.1046177>

Lindsey, M. A., Brandt, N. E., Becker, K. D., Lee, B. R., Barth, R. P., Daleiden, E. L., & Chorpita, B. F. (2014). Identifying the common elements of treatment engagement interventions in children’s mental health services. *Clinical Child and Family Psychology Review*, *17*(3), 283–298.<https://doi.org/10.1007/s10567-013-0163-x>

Ng, M. Y., Eckshtain, D., & Weisz, J. R. (2016). Assessing Fit Between Evidence-Based Psychotherapies for Youth Depression and Real-Life Coping in Early Adolescence. *Journal of Clinical Child and Adolescent Psychology: The Official Journal for the Society of Clinical Child and Adolescent Psychology, American Psychological Association, Division 53*, *45*(6), 732–748.<https://doi.org/10.1080/15374416.2015.1041591>

Strosahl, K. D., & Robinson, P. J. (2017). *The Mindfulness and Acceptance Workbook for Depression: Using Acceptance and Commitment Therapy to Move Through Depression and Create a Life Worth Living*. New Harbinger Publications.
