# Supplementary material for: Commonly Reported Problems and Coping Strategies During the COVID-19 Crisis: A Survey of Graduate and Professional Students
Source: Front Psychol. 2021 Feb 25;12:598557. doi: 10.3389/fpsyg.2021.598557 (PMC7947789; doi:10.3389/fpsyg.2021.598557)
Supplement: Supplementary file 2 [file Table_2.DOCX]

**Supplementary File Two: Full List of Strategies and Frequencies**

Table S1

*Common and Effective Strategies Endorsed by Graduate Students*

| Strategies | Percentage of People Endorsing the Strategy as a Common Strategy | Percentage of People Endorsing the Strategy as an Effective Strategy |
| --- | --- | --- |
| EBP (distraction included) | 85% (n = 257) | 89% (n = 271) |
| EBP (distraction excluded) | 42% (128) | 74% (226) |
| **Distraction** | **43% (131)** | **15% (45)** |
| Behavioral | 42% (126) | 14% (43) |
| TV | 18% (55) | 3% (10) |
| Food | 9% (28) | 1% (3) |
| Productivity | 4% (13) | 7% (20) |
| Social Media | 3% (10) | 0% (0) |
| Reading | 3% (9) | 2% (5) |
| Music | 2% (6) | 0% (1) |
| Cognitive | 0% (1) | 1% (2) |
| **Behavioral Activation** | **27% (83)** | **50% (152)** |
| Physical Activity | 19% (57) | 40% (121) |
| Going Outside | 5% (15) | 14% (43) |
| Social Activities | 3% (8) | 4% (12) |
| Routine | 1% (2) | 2% (7) |
| **Increasing Social Support** | **9% (26)** | **12% (37)** |
| Friend | 5% (15) | 9% (26) |
| Family Member | 3% (8) | 4% (13) |
| Significant Other | 1% (3) | 2% (7) |
| Help | 0% (0) | 1% (3) |
| Feelings | 0% (0) | 1% (3) |
| Refraining from Activity | 5% (16) | 1% (3) |
| Sleep | 3% (8) | 1% (2) |
| Miscellaneous | 3% (8) | 3% (9) |
| Mindfulness/Meditation | 2% (6) | 5% (14) |
| Information Seeking | 2% (6) | 0% (0) |
| Problem Solving | 1% (4) | 2% (5) |
| Substance Use | 1% (4) | 0% (0) |
| Avoidance | 1% (3) | 1% (3) |
| Relaxation | 1% (4) | 3% (10) |
| Nothing | 1% (3) | 0% (1) |
| Reframing | 1% (3) | 1% (4) |
| Goal Setting | 1% (2) | 1% (4) |
| Religion | 1% (2) | 3% (8) |
| Gratitude | 0% (1) | 1% (2) |
| Non-communicative Expression of Feelings | 1% (2) | 1% (3) |
| Crying | 1% (2) | 1% (3) |
| Externalizing Behavior | 0% (0) | 0% (0) |
| Journaling | 0% (1) | 1% (2) |
| Identifying Stress-Related Situations | 0% (0) | 0% (0) |
| Avoiding News | 0% (1) | 2% (6) |
| Humor | 0% (0) | 0% (0) |
| Practice or Perseverance | 0% (0) | 0% (0) |
| Modeling | 0% (0) | 0% (0) |
| Self-Monitoring | 0% (0) | 0% (0) |
| Psychoeducation | 0% (0) | 0% (0) |
| Reinforcement | 0% (0) | 0% (0) |
| Understanding Affect | 0% (1) | 1% (4) |
| Understanding and Finding Meaning in Loss and Change | 0% (1) | 0% (0) |
| Personalizing Treatment | 0% (0) | 0% (0) |
| Building a Positive Sense of Self | 0% (0) | 0% (0) |
| Understanding Relationships and their Link to Affect | 0% (0) | 0% (0) |
| Limited Sick Role | 0% (0) | 0% (0) |
| Exposure | 0% (0) | 0% (0) |
| Guided Imagery | 0% (0) | 0% (0) |
| Values | 0% (0) | 0% (0) |
| Kindness to Self | 0% (0) | 0% (1) |
| Kindness to Others | 0% (0) | 0% (0) |
|  |  |  |
