# Supplementary material for: Commonly Reported Problems and Coping Strategies During the COVID-19 Crisis: A Survey of Graduate and Professional Students
Source: Front Psychol. 2021 Feb 25;12:598557. doi: 10.3389/fpsyg.2021.598557 (PMC7947789; doi:10.3389/fpsyg.2021.598557)
Supplement: Supplementary file 3 [file Table_3.DOCX]

# August 14, 2020

# Akash R. Wasil and Rose E. Franzen

# Commonly Reported Problems and Coping Strategies During the COVID-19 Crisis: A Survey of Graduate and Professional Students

# In this file, we present our analyses in the order that they appear in the manuscript.

# Please email wasil@sas.upenn.edu with any questions.

#Notes about variable notation:

##Variables that start with "Com_" refer to strategies reported as common

##Variables that start with "Eff_" refer to strategies reported as effective

# Loading packages

if (!require("tm")) {install.packages("tm"); require("tm")}

if (!require("psych")) {install.packages("psych"); require("psych")}

if (!require("vcd")) {install.packages("vcd"); require("vcd")}

if (!require("effsize")) {install.packages("effsize"); require("effsize")}

if (!require(ggplot2)) {install.packages("ggplot2"); require(ggplot2)}

if (!require(psych)) {install.packages("psych"); require(psych)}

if (!require(lmerTest)) {install.packages("lmerTest"); require(lmerTest)} # uses lme4 package, gives p-values for regression parameters

if (!require(nlme)) {install.packages("nlme"); require(nlme)}

if (!require(reshape)) {install.packages("reshape"); require(reshape)}

if (!require(lattice)) {install.packages("lattice"); require(lattice)}

if (!require(car)) {install.packages('car'); require(car)}

if (!require(effects)) {install.packages("effects"); require(effects)}

if (!require("sjPlot")) {install.packages("sjPlot"); require("sjPlot")}

if (!require("mice")) {install.packages("mice"); require("mice")} # for imputation of missing values

if (!require(plyr)) {install.packages("plyr"); require(plyr)} # for count function

library(mediation)

library(beanplot)

library(mgcv)

if(!require(ltm)) install.packages("ltm"); library("ltm")

if(!require(ggpubr)) install.packages("ggpubr"); library("ggpubr")

# Loading dataset, recoding variables, and computing sums

Coping<- read.csv("Problems and Coping Data.csv")

Coping$Age<- as.numeric(as.character(Coping$Age))

Coping$Com_EBP<- as.factor(as.character(Coping$Com_EBP))

Coping$Eff_EBP<- as.factor(as.character(Coping$Eff_EBP))

Coping$Com_EBP_NoDist<- as.factor(as.character(Coping$Com_EBP_NoDist))

Coping$Eff_EBP_NoDist<- as.factor(as.character(Coping$Eff_EBP_NoDist))

Coping$PHQ.Total<- Coping$PHQ_1+Coping$PHQ_2

Coping$GAD.Total<- Coping$GAD_1+Coping$GAD_2

#Method

##Calculating Cronbach's alphas

PHQMat<- cbind(Coping$PHQ_1, Coping$PHQ_2)

GADMat<- cbind(Coping$GAD_1, Coping$GAD_2)

psych::alpha(PHQMat) #0.8

psych::alpha(GADMat) #0.86

#Results

##Sample Characteristics/Table 1

###PHQ

mean(Coping$PHQ.Total) # mean = 2.04

sd(Coping$PHQ.Total) # SD = 1.69

###GAD

mean(Coping$GAD.Total) # mean = 2.68

sd(Coping$GAD.Total) # 1.87

###Age

describe(Coping$Age) # mean = 31.04, SD = 8.91 N = 169; 136 missing

###Sex

plyr::count(Coping$Sex)

127/(127+42+3+2) # 72.99% female

42/(127+42+3+2) # 24.14% male

3/(127+42+3+2)# 1.72% Prefer not to answer

2/(127+42+3+2) # 1.15% Other

# 131 missing

###Race

plyr::count(Coping$Race)

(104+3+5+2)/(37+1+3+9+1+6+5+1+2+2+104) # 66.67% any White

(37+1+3)/(37+1+3+9+1+6+5+1+2+2+104) # 23.98% any Asian

(1+9+1)/(37+1+3+9+1+6+5+1+2+2+104) # 6.43% any Black

(1+6+5)/(37+1+3+9+1+6+5+1+2+2+104) # 7.02% any Hispanic

(1+2)/(37+1+3+9+1+6+5+1+2+2+104) # 1.75% any Middle Eastern/North African

(2)/(37+1+3+9+1+6+5+1+2+2+104) # 1.17% any other

# 134 missing

###Sexual Orientation

plyr::count(Coping$Sexuality)

(1+1+1+1)/(172) # 2.33% Asexual

(9+1+2+2+1+1)/172 # 9.30% Bisexual

(1+1+1)/172 # 1.74% Demisexual

(1+2+1+1+1)/172 # 3.49% Fluid

(2+2+1)/172 # 2.91% Gay or Lesbian

(136+1+1+1+1)/172 # 81.40% Heterosexual/Straight

(2+1+1+1)/172 # 2.91% Pansexual

(1+2+2+2+1+2)/172 # 5.81% Queer

(1+1+1)/172 # 1.74% Questioning

5/172 # 2.91% Prefer not to answer

# 133 missing

###Social Class

plyr::count(Coping$Economic.Class)

30/173*100 # 17.34% Affluent

111/173*100 # 64.16% Middle class

5/173*100 # Poor 2.89%

27/173*100 # Working class 15.61%

# 132 missing

###Personal Experience with Mental Illness

plyr::count(Coping$Past.Experience)

79/173 # 45.67% No

22/173 # 12.72% Unsure

72/173 # 41.62% Yes

# 132 missing

## McNemar Tests

###Creating "Other" category to lump together all codes not endorsed by at least 5%

Coping$Eff_Other<- NA

Coping$Com_Other<- NA

for (i in 1:length(Coping$ResponseId)) {

if(Coping$Eff_BA[i] ==0 & Coping$Eff_Distraction[i] ==0 & Coping$Eff_Social.Support[i]==0) Coping$Eff_Other[i] <- 1

else(Coping$Eff_Other[i] <- 0)

if(Coping$Com_BA[i] ==0& Coping$Com_Distraction[i]==0 & Coping$Com_Social.Support[i]==0) Coping$Com_Other[i] <- 1

else(Coping$Com_Other[i] <- 0)

}

###BA Vector

sum(Coping$Com_BA==1, na.rm = TRUE) # 83

sum(Coping$Com_BA==1&Coping$Eff_BA==1, na.rm=TRUE) # 55

sum(Coping$Com_BA==1&Coping$Eff_Distraction==1, na.rm = TRUE) # 7

sum(Coping$Com_BA==1&Coping$Eff_Social.Support==1, na.rm = TRUE) # 7

sum(Coping$Com_BA==1&Coping$Eff_Other==1, na.rm = TRUE) # 14

BAVec<- c(55, 7, 7, 14)

###Distraction Vector

sum(Coping$Com_Distraction==1, na.rm = TRUE) # 131

sum(Coping$Com_Distraction==1&Coping$Eff_BA==1, na.rm = TRUE) # 58

sum(Coping$Com_Distraction==1&Coping$Eff_Distraction==1, na.rm = TRUE) # 29

sum(Coping$Com_Distraction==1&Coping$Eff_Social.Support==1, na.rm = TRUE) # 17

sum(Coping$Com_Distraction==1&Coping$Eff_Other==1, na.rm = TRUE) # 27

DistractVec<- c(58, 29, 17, 27)

###Social Support Vector

sum(Coping$Com_Social.Support==1, na.rm = TRUE) # 26

sum(Coping$Com_Social.Support==1&Coping$Eff_BA==1, na.rm = TRUE) # 14

sum(Coping$Com_Social.Support==1&Coping$Eff_Distraction==1, na.rm = TRUE) # 2

sum(Coping$Com_Social.Support==1&Coping$Eff_Social.Support==1, na.rm = TRUE) # 6

sum(Coping$Com_Social.Support==1&Coping$Eff_Other==1, na.rm = TRUE) # 4

SSVec<- c(14, 2, 6, 4)

###Other Vector

sum(Coping$Com_Other==1, na.rm = TRUE) # 67

sum(Coping$Com_Other==1&Coping$Eff_BA==1, na.rm = TRUE) # 26

sum(Coping$Com_Other==1&Coping$Eff_Distraction==1, na.rm = TRUE) # 7

sum(Coping$Com_Other==1&Coping$Eff_Social.Support==1, na.rm = TRUE) # 7

sum(Coping$Com_Other==1&Coping$Eff_Other==1, na.rm = TRUE) # 27

OtherVec<- c(26, 7, 7, 27)

###McNemar-Bowker Omnibus

McNemarMat<- rbind(BAVec, DistractVec, SSVec, OtherVec)

mcnemar.test(McNemarMat, correct = FALSE) # p = 0.0000000000003

###Post-Hoc Tests

####BA and Distraction

sum(Coping$Com_BA==1&Coping$Eff_BA==1, na.rm = TRUE) # 55

sum(Coping$Com_BA==1&Coping$Eff_Distraction==1) #7

sum(Coping$Com_Distraction==1&Coping$Eff_BA==1) #58

sum(Coping$Com_Distraction==1&Coping$Eff_Distraction==1, na.rm = TRUE) #29

BADist1<- c(55, 7)

BADist2<- c(58, 29)

BADistMat<- rbind(BADist1, BADist2)

mcnemar.test(BADistMat, correct = FALSE) # p = 0.0000000003

##BA and SS

sum(Coping$Com_BA==1&Coping$Eff_BA==1, na.rm = TRUE) # 55

sum(Coping$Com_BA==1&Coping$Eff_Social.Support==1) # 7

sum(Coping$Com_Social.Support==1&Coping$Eff_BA==1) # 14

sum(Coping$Com_Social.Support==1&Coping$Eff_Social.Support==1, na.rm = TRUE) # 6

BASS1<- c(55, 7)

BASS2<- c(14, 6)

BASSMat<- rbind(BASS1, BASS2)

mcnemar.test(BASSMat, correct = FALSE) # p = 0.13

##BA and Other

sum(Coping$Com_BA==1&Coping$Eff_BA==1, na.rm = TRUE) # 55

sum(Coping$Com_BA==1&Coping$Eff_Other==1) # 14

sum(Coping$Com_Other==1&Coping$Eff_BA==1) # 26

sum(Coping$Com_Other==1&Coping$Eff_Other==1, na.rm = TRUE) # 27

BAOther1<- c(55, 14)

BAOther2<- c(26, 27)

BAOtherMat<- rbind(BAOther1, BAOther2)

mcnemar.test(BAOtherMat, correct = FALSE) # p = 0.06

##Distraction and Social Support

sum(Coping$Com_Distraction==1&Coping$Eff_Distraction==1, na.rm = TRUE) # 29

sum(Coping$Com_Distraction==1&Coping$Eff_Social.Support==1) # 17

sum(Coping$Com_Social.Support==1&Coping$Eff_Distraction==1) # 2

sum(Coping$Com_Social.Support==1&Coping$Eff_Social.Support==1, na.rm = TRUE) # 6

DistSS1<- c(29, 17)

DistSS2<- c(2, 6)

DistSSMat<- rbind(DistSS1, DistSS2)

mcnemar.test(DistSSMat, correct = FALSE) # p = 0.0006

##Distraction and Other

sum(Coping$Com_Distraction==1&Coping$Eff_Distraction==1, na.rm = TRUE) # 29

sum(Coping$Com_Distraction==1&Coping$Eff_Other==1) # 27

sum(Coping$Com_Other==1&Coping$Eff_Distraction==1) # 7

sum(Coping$Com_Other==1&Coping$Eff_Other==1, na.rm = TRUE) # 27

DistOther1<- c(29, 27)

DistOther2<- c(7, 27)

DistOtherMat<- rbind(DistOther1, DistOther2)

mcnemar.test(DistOtherMat, correct = FALSE) # p = 0.0006

##Social Support and Other

sum(Coping$Com_Social.Support==1&Coping$Eff_Social.Support==1, na.rm = TRUE) # 6

sum(Coping$Com_Social.Support==1&Coping$Eff_Other==1) # 4

sum(Coping$Com_Other==1&Coping$Eff_Social.Support==1) # 7

sum(Coping$Com_Other==1&Coping$Eff_Other==1, na.rm = TRUE) # 27

SSOther1<- c(6, 4)

SSOther2<- c(7, 27)

SSOtherMat<- rbind(SSOther1, SSOther2)

mcnemar.test(SSOtherMat, correct = FALSE) # p = 0.37

## EBP Frequencies

count(Coping$Eff_EBP) # 271 EBP, 33 non-EBP; 1 missing (did not provide a response for Effective strategy)

271/(271+32) # 89.44% EBP

count(Coping$Com_EBP) # 257 EBP, 46 non-EBP; 2 missing (did not provide a response for Common strategy)

257/(257+46) # 84.82% EBP

### distraction NOT considered an EBP

count(Coping$Eff_EBP_NoDist) # 226 EBP, 78 non-EBP; 1 missing

226/(226+78) # 74.34% EBP

count(Coping$Com_EBP_NoDist) # 128 EBP, 175 non-EBP; 2 missing

128/(128+175) # 42.24% EBP

##Associations between EBPs and Mental Health

###Checking variance to ensure meets assumptions for t-tests

var.test(Coping$PHQ.Total[Coping$Eff_EBP==1], Coping$PHQ.Total[Coping$Eff_EBP==0])

var.test(Coping$GAD.Total[Coping$Eff_EBP==1], Coping$GAD.Total[Coping$Eff_EBP==0])

var.test(Coping$PHQ.Total[Coping$Eff_EBP_NoDist==1], Coping$PHQ.Total[Coping$Eff_EBP_NoDist==0])

var.test(Coping$GAD.Total[Coping$Eff_EBP_NoDist==1], Coping$GAD.Total[Coping$Eff_EBP_NoDist==0])

var.test(Coping$PHQ.Total[Coping$Com_EBP==1], Coping$PHQ.Total[Coping$Com_EBP==0]) # unequal variances

var.test(Coping$GAD.Total[Coping$Com_EBP==1], Coping$GAD.Total[Coping$Com_EBP==0]) # unequal variances

var.test(Coping$PHQ.Total[Coping$Com_EBP_NoDist==1], Coping$PHQ.Total[Coping$Com_EBP_NoDist==0]) # unequal variances

var.test(Coping$GAD.Total[Coping$Com_EBP_NoDist==1], Coping$GAD.Total[Coping$Com_EBP_NoDist==0])

###EBPs as Effective Strategies & PHQ

####Endorsing

mean(Coping$PHQ.Total[Coping$Eff_EBP==1], na.rm = TRUE) # mean = 2.00

sd(Coping$PHQ.Total[Coping$Eff_EBP==1], na.rm = TRUE) # SD = 1.65

####Not endorsing

mean(Coping$PHQ.Total[Coping$Eff_EBP==0], na.rm = TRUE) # mean = 2.30

sd(Coping$PHQ.Total[Coping$Eff_EBP==0], na.rm = TRUE) # SD = 2.01

t.test(Coping$PHQ.Total[Coping$Eff_EBP==1],

Coping$PHQ.Total[Coping$Eff_EBP==0], alternative = "less", var.equal = TRUE) # p = 0.17

cohen.d(Coping$PHQ.Total[Coping$Eff_EBP==1],

Coping$PHQ.Total[Coping$Eff_EBP==0], na.rm = TRUE) # d = 0.18 (raw d value is negative, reporting as positive to indicate better mental health outcome)

### EBPs as Effective Strategies & GAD

####Endorsing

mean(Coping$GAD.Total[Coping$Eff_EBP==1], na.rm = TRUE) # mean = 2.69

sd(Coping$GAD.Total[Coping$Eff_EBP==1], na.rm = TRUE) # SD = 1.88

####Not endorsing

mean(Coping$GAD.Total[Coping$Eff_EBP==0], na.rm = TRUE)# mean = 2.67

sd(Coping$GAD.Total[Coping$Eff_EBP==0], na.rm = TRUE) # SD = 1.83

t.test(Coping$GAD.Total[Coping$Eff_EBP==1],

Coping$GAD.Total[Coping$Eff_EBP==0], alternative = "less", var.equal = TRUE) # p = 0.52

cohen.d(Coping$GAD.Total[Coping$Eff_EBP==1],

Coping$GAD.Total[Coping$Eff_EBP==0], na.rm = TRUE) # d = -0.01

###EBPs (distraction excluded) as Effective Strategies & PHQ

####Endorsing

mean(Coping$PHQ.Total[Coping$Eff_EBP_NoDist==1], na.rm = TRUE) # mean = 1.96

sd(Coping$PHQ.Total[Coping$Eff_EBP_NoDist==1], na.rm = TRUE) # SD = 1.66

####Not endorsing

mean(Coping$PHQ.Total[Coping$Eff_EBP_NoDist==0], na.rm = TRUE) # mean = 2.26

sd(Coping$PHQ.Total[Coping$Eff_EBP_NoDist==0], na.rm = TRUE) # SD = 1.76

t.test(Coping$PHQ.Total[Coping$Eff_EBP_NoDist==1],

Coping$PHQ.Total[Coping$Eff_EBP_NoDist==0], alternative = "less", var.equal = TRUE) # p = 0.09

cohen.d(Coping$PHQ.Total[Coping$Eff_EBP_NoDist==1],

Coping$PHQ.Total[Coping$Eff_EBP_NoDist==0], na.rm = TRUE) # d = 0.18

### EBPs (distraction excluded) as Effective Strategies & GAD

####Endorsing

mean(Coping$GAD.Total[Coping$Eff_EBP_NoDist==1], na.rm = TRUE) # mean = 2.72

sd(Coping$GAD.Total[Coping$Eff_EBP_NoDist==1], na.rm = TRUE) # SD = 1.88

####Not endorsing

mean(Coping$GAD.Total[Coping$Eff_EBP_NoDist==0], na.rm = TRUE)# mean = 2.58

sd(Coping$GAD.Total[Coping$Eff_EBP_NoDist==0], na.rm = TRUE) # SD = 1.87

t.test(Coping$GAD.Total[Coping$Eff_EBP_NoDist==1],

Coping$GAD.Total[Coping$Eff_EBP_NoDist==0], alternative = "less", var.equal = TRUE) # p = 0.72

cohen.d(Coping$GAD.Total[Coping$Eff_EBP_NoDist==1],

Coping$GAD.Total[Coping$Eff_EBP_NoDist==0], na.rm = TRUE) # d = -0.08

###EBPs as Common Strategies & PHQ

####Endorsing

mean(Coping$PHQ.Total[Coping$Com_EBP==1], na.rm = TRUE) # mean = 1.88

sd(Coping$PHQ.Total[Coping$Com_EBP==1], na.rm = TRUE) # SD = 1.55

####Not endorsing

mean(Coping$PHQ.Total[Coping$Com_EBP==0], na.rm = TRUE) # mean = 2.91

sd(Coping$PHQ.Total[Coping$Com_EBP==0], na.rm = TRUE) # SD = 2.17

t.test(Coping$PHQ.Total[Coping$Com_EBP==1],

Coping$PHQ.Total[Coping$Com_EBP==0], alternative = "less",) # p = 0.002

cohen.d(Coping$PHQ.Total[Coping$Com_EBP==1],

Coping$PHQ.Total[Coping$Com_EBP==0], na.rm = TRUE) # d = 0.62

###EBPs as Common Strategies & GAD

####Endorsing

mean(Coping$GAD.Total[Coping$Com_EBP==1], na.rm = TRUE) # mean = 2.53

sd(Coping$GAD.Total[Coping$Com_EBP==1], na.rm = TRUE) # SD = 1.77

####Not endorsing

mean(Coping$GAD.Total[Coping$Com_EBP==0], na.rm = TRUE)# mean = 3.54

sd(Coping$GAD.Total[Coping$Com_EBP==0], na.rm = TRUE) # SD = 2.20

t.test(Coping$GAD.Total[Coping$Com_EBP==1],

Coping$GAD.Total[Coping$Com_EBP==0], alternative = "less") # p = 0.002

cohen.d(Coping$GAD.Total[Coping$Com_EBP==1],

Coping$GAD.Total[Coping$Com_EBP==0], na.rm = TRUE) # d = 0.55

###EBPs (distraction excluded) as Common Strategies & PHQ

####Endorsing

mean(Coping$PHQ.Total[Coping$Com_EBP_NoDist==1], na.rm = TRUE) # mean = 1.63

sd(Coping$PHQ.Total[Coping$Com_EBP_NoDist==1], na.rm = TRUE) # SD = 1.47

####Not endorsing

mean(Coping$PHQ.Total[Coping$Com_EBP_NoDist==0], na.rm = TRUE) # mean = 2.34

sd(Coping$PHQ.Total[Coping$Com_EBP_NoDist==0], na.rm = TRUE) # SD = 1.78

t.test(Coping$PHQ.Total[Coping$Com_EBP_NoDist==1],

Coping$PHQ.Total[Coping$Com_EBP_NoDist==0], alternative = "less") # p = 0.00008

cohen.d(Coping$PHQ.Total[Coping$Com_EBP_NoDist==1],

Coping$PHQ.Total[Coping$Com_EBP_NoDist==0], na.rm = TRUE) # d = 0.43

###EBPs (distraction excluded) as Common Strategies & GAD

####Endorsing

mean(Coping$GAD.Total[Coping$Com_EBP_NoDist==1], na.rm = TRUE) # mean = 2.37

sd(Coping$GAD.Total[Coping$Com_EBP_NoDist==1], na.rm = TRUE) # SD = 1.69

####Not endorsing

mean(Coping$GAD.Total[Coping$Com_EBP_NoDist==0], na.rm = TRUE) # mean = 2.92

sd(Coping$GAD.Total[Coping$Com_EBP_NoDist==0], na.rm = TRUE) # SD = 1.97

t.test(Coping$GAD.Total[Coping$Com_EBP_NoDist==1],

Coping$GAD.Total[Coping$Com_EBP_NoDist==0], alternative = "less", var.equal = TRUE) # p = 0.006

cohen.d(Coping$GAD.Total[Coping$Com_EBP_NoDist==1],

Coping$GAD.Total[Coping$Com_EBP_NoDist==0], na.rm = TRUE) # d = 0.30

##Assocations between Matching Common/Effective and Mental Health

###Checking variance to ensure meets assumptions for t-tests

var.test(Coping$PHQ.Total[Coping$Match==1], Coping$PHQ.Total[Coping$Match==0])

var.test(Coping$GAD.Total[Coping$Match==1], Coping$GAD.Total[Coping$Match==0])

###Match & PHQ

####Matchers

mean(Coping$PHQ.Total[Coping$Match==1], na.rm = TRUE) # mean = 1.64

sd(Coping$PHQ.Total[Coping$Match==1], na.rm = TRUE) # SD = 1.46

####Non-matchers

mean(Coping$PHQ.Total[Coping$Match==0], na.rm = TRUE) # mean = 2.20

sd(Coping$PHQ.Total[Coping$Match==0], na.rm = TRUE) # SD = 1.76

t.test(Coping$PHQ.Total[Coping$Match==1],

Coping$PHQ.Total[Coping$Match==0], alternative = "less", var.equal = TRUE) # p = 0.005

cohen.d(Coping$PHQ.Total[Coping$Match==1],

Coping$PHQ.Total[Coping$Match==0], na.rm = TRUE) # d = 0.33

###Match & GAD

####Matchers

mean(Coping$GAD.Total[Coping$Match==1], na.rm = TRUE) # mean = 2.53

sd(Coping$GAD.Total[Coping$Match==1], na.rm = TRUE) # SD = 1.73

####Non-matchers

mean(Coping$GAD.Total[Coping$Match==0], na.rm = TRUE) # mean = 2.75

sd(Coping$GAD.Total[Coping$Match==0], na.rm = TRUE) # SD = 1.93

t.test(Coping$GAD.Total[Coping$Match==1],

Coping$GAD.Total[Coping$Match==0], alternative = "less", var.equal = TRUE) # p = 0.18

cohen.d(Coping$GAD.Total[Coping$Match==1],

Coping$GAD.Total[Coping$Match==0], na.rm = TRUE) # d = 0.12

##Associations between Specific Strategies and Mental Health

###Behavioral Activation

####Checking variance to ensure meets assumptions for t-tests

var.test(Coping$PHQ.Total[Coping$Com_BA==1], Coping$PHQ.Total[Coping$Com_BA==0])

var.test(Coping$GAD.Total[Coping$Com_BA==1], Coping$GAD.Total[Coping$Com_BA==0])

var.test(Coping$PHQ.Total[Coping$Eff_BA==1], Coping$PHQ.Total[Coping$Eff_BA==0])

var.test(Coping$GAD.Total[Coping$Eff_BA==1], Coping$GAD.Total[Coping$Eff_BA==0])

####Common strategy & PHQ

#####Endorsers

mean(Coping$PHQ.Total[Coping$Com_BA==1], na.rm = TRUE) # mean = 1.59

sd(Coping$PHQ.Total[Coping$Com_BA==1], na.rm = TRUE) # SD = 1.52

#####Non-Endorsers

mean(Coping$PHQ.Total[Coping$Com_BA==0], na.rm = TRUE) # mean = 2.20

sd(Coping$PHQ.Total[Coping$Com_BA==0], na.rm = TRUE) # SD = 1.72

t.test(Coping$PHQ.Total[Coping$Com_BA==1], Coping$PHQ.Total[Coping$Com_BA==0], var.equal = TRUE) # p = 0.005

cohen.d(Coping$PHQ.Total[Coping$Com_BA==1], Coping$PHQ.Total[Coping$Com_BA==0], na.rm = TRUE) # d = 0.37

####Common strategy & GAD

#####Endorsers

mean(Coping$GAD.Total[Coping$Com_BA==1], na.rm = TRUE) # mean = 2.31

sd(Coping$GAD.Total[Coping$Com_BA==1], na.rm = TRUE) # SD = 1.70

#####Non-Endorsers

mean(Coping$GAD.Total[Coping$Com_BA==0], na.rm = TRUE) # mean = 2.82

sd(Coping$GAD.Total[Coping$Com_BA==0], na.rm = TRUE) # SD = 1.92

t.test(Coping$GAD.Total[Coping$Com_BA==1], Coping$GAD.Total[Coping$Com_BA==0], var.equal = TRUE) # p = 0.04

cohen.d(Coping$GAD.Total[Coping$Com_BA==1], Coping$GAD.Total[Coping$Com_BA==0], na.rm = TRUE) # d = 0.27

####Effective strategy & PHQ

#####Endorsers

mean(Coping$PHQ.Total[Coping$Eff_BA==1], na.rm = TRUE) # mean = 2.09

sd(Coping$PHQ.Total[Coping$Eff_BA==1], na.rm = TRUE) # SD = 1.72

####Non-Endorsers

mean(Coping$PHQ.Total[Coping$Eff_BA==0], na.rm = TRUE) # mean = 1.98

sd(Coping$PHQ.Total[Coping$Eff_BA==0], na.rm = TRUE) # SD = 1.66

t.test(Coping$PHQ.Total[Coping$Eff_BA==1], Coping$PHQ.Total[Coping$Eff_BA==0], var.equal = TRUE) # p = 0.56

cohen.d(Coping$PHQ.Total[Coping$Eff_BA==1], Coping$PHQ.Total[Coping$Eff_BA==0], na.rm = TRUE) # d = -0.07

####Effective Strategy & GAD

#####Endorsers

mean(Coping$GAD.Total[Coping$Eff_BA==1], na.rm = TRUE) # mean = 2.81

sd(Coping$GAD.Total[Coping$Eff_BA==1], na.rm = TRUE) # SD = 1.92

#####Non-Endorsers

mean(Coping$GAD.Total[Coping$Eff_BA==0], na.rm = TRUE) # mean = 2.56

sd(Coping$GAD.Total[Coping$Eff_BA==0], na.rm = TRUE) # SD = 1.81

t.test(Coping$GAD.Total[Coping$Eff_BA==1], Coping$GAD.Total[Coping$Eff_BA==0], var.equal = TRUE) # p = 0.24

cohen.d(Coping$GAD.Total[Coping$Eff_BA==1], Coping$GAD.Total[Coping$Eff_BA==0], na.rm = TRUE) # d = -0.14

###Distraction

####Checking variance to ensure meets assumptions for t-tests

var.test(Coping$PHQ.Total[Coping$Com_Distraction==1], Coping$PHQ.Total[Coping$Com_Distraction==0])

var.test(Coping$GAD.Total[Coping$Com_Distraction==1], Coping$GAD.Total[Coping$Com_Distraction==0])

var.test(Coping$PHQ.Total[Coping$Eff_Distraction==1], Coping$PHQ.Total[Coping$Eff_Distraction==0])

var.test(Coping$GAD.Total[Coping$Eff_Distraction==1], Coping$GAD.Total[Coping$Eff_Distraction==0])

####Common strategy & PHQ

#####Endorsers

mean(Coping$PHQ.Total[Coping$Com_Distraction==1], na.rm = TRUE) # mean = 2.11

sd(Coping$PHQ.Total[Coping$Com_Distraction==1], na.rm = TRUE) # SD = 1.58

#####Non-Endorsers

mean(Coping$PHQ.Total[Coping$Com_Distraction==0], na.rm = TRUE) # mean = 1.98

sd(Coping$PHQ.Total[Coping$Com_Distraction==0], na.rm = TRUE) # SD = 1.77

t.test(Coping$PHQ.Total[Coping$Com_Distraction==1], Coping$PHQ.Total[Coping$Com_Distraction==0], var.equal = TRUE) # p = 0.48

cohen.d(Coping$PHQ.Total[Coping$Com_Distraction==1], Coping$PHQ.Total[Coping$Com_Distraction==0], na.rm = TRUE) # d = -0.08

####Common strategy & GAD

#####Endorsers

mean(Coping$GAD.Total[Coping$Com_Distraction==1], na.rm = TRUE) # mean = 2.68

sd(Coping$GAD.Total[Coping$Com_Distraction==1], na.rm = TRUE) # SD = 1.84

#####Non-Endorsers

mean(Coping$GAD.Total[Coping$Com_Distraction==0], na.rm = TRUE) # mean = 2.68

sd(Coping$GAD.Total[Coping$Com_Distraction==0], na.rm = TRUE) # SD = 1.90

t.test(Coping$GAD.Total[Coping$Com_Distraction==1], Coping$GAD.Total[Coping$Com_Distraction==0], var.equal = TRUE) # p = 0.98

cohen.d(Coping$GAD.Total[Coping$Com_Distraction==1], Coping$GAD.Total[Coping$Com_Distraction==0], na.rm = TRUE) # d = -0.002

####Effective strategy & PHQ

#####Endorsers

mean(Coping$PHQ.Total[Coping$Eff_Distraction==1], na.rm = TRUE) # mean = 2.22

sd(Coping$PHQ.Total[Coping$Eff_Distraction==1], na.rm = TRUE) # SD = 1.58

#####Non-Endorsers

mean(Coping$PHQ.Total[Coping$Eff_Distraction==0], na.rm = TRUE) # mean = 2.00

sd(Coping$PHQ.Total[Coping$Eff_Distraction==0], na.rm = TRUE) # SD = 1.71

t.test(Coping$PHQ.Total[Coping$Eff_Distraction==1], Coping$PHQ.Total[Coping$Eff_Distraction==0], var.equal = TRUE) # p = 0.42

cohen.d(Coping$PHQ.Total[Coping$Eff_Distraction==1], Coping$PHQ.Total[Coping$Eff_Distraction==0], na.rm = TRUE) # d = -0.13

####Effective Strategy & GAD

#####Endorsers

mean(Coping$GAD.Total[Coping$Eff_Distraction==1], na.rm = TRUE) # mean = 2.51

sd(Coping$GAD.Total[Coping$Eff_Distraction==1], na.rm = TRUE) # SD = 1.91

#####Non-Endorsers

mean(Coping$GAD.Total[Coping$Eff_Distraction==0], na.rm = TRUE) # mean = 2.71

sd(Coping$GAD.Total[Coping$Eff_Distraction==0], na.rm = TRUE) # SD = 1.87

t.test(Coping$GAD.Total[Coping$Eff_Distraction==1], Coping$GAD.Total[Coping$Eff_Distraction==0], var.equal = TRUE) # p = 0.51

cohen.d(Coping$GAD.Total[Coping$Eff_Distraction==1], Coping$GAD.Total[Coping$Eff_Distraction==0], na.rm = TRUE) # d = 0.11

###Television

####Checking variance to ensure meets assumptions for t-tests

var.test(Coping$PHQ.Total[Coping$Com_TV==1], Coping$PHQ.Total[Coping$Com_TV==0])

var.test(Coping$GAD.Total[Coping$Com_TV==1], Coping$GAD.Total[Coping$Com_TV==0])

var.test(Coping$PHQ.Total[Coping$Eff_TV==1], Coping$PHQ.Total[Coping$Eff_TV==0])

var.test(Coping$GAD.Total[Coping$Eff_TV==1], Coping$GAD.Total[Coping$Eff_TV==0])

####Common strategy & PHQ

#####Endorsers

mean(Coping$PHQ.Total[Coping$Com_TV==1], na.rm = TRUE) # mean = 2.11

sd(Coping$PHQ.Total[Coping$Com_TV==1], na.rm = TRUE) # SD = 1.57

#####Non-Endorsers

mean(Coping$PHQ.Total[Coping$Com_TV==0], na.rm = TRUE) # mean = 2.02

sd(Coping$PHQ.Total[Coping$Com_TV==0], na.rm = TRUE) # SD = 1.72

t.test(Coping$PHQ.Total[Coping$Com_TV==1], Coping$PHQ.Total[Coping$Com_TV==0], var.equal = TRUE) # p = 0.72

cohen.d(Coping$PHQ.Total[Coping$Com_TV==1], Coping$PHQ.Total[Coping$Com_TV==0], na.rm = TRUE) # d = -0.05

####Common strategy & GAD

#####Endorsers

mean(Coping$GAD.Total[Coping$Com_TV==1], na.rm = TRUE) # mean = 2.58

sd(Coping$GAD.Total[Coping$Com_TV==1], na.rm = TRUE) # SD = 1.72

#####Non-Endorsers

mean(Coping$GAD.Total[Coping$Com_TV==0], na.rm = TRUE) # mean = 2.70

sd(Coping$GAD.Total[Coping$Com_TV==0], na.rm = TRUE) # SD = 1.91

t.test(Coping$GAD.Total[Coping$Com_TV==1], Coping$GAD.Total[Coping$Com_TV==0], var.equal = TRUE) # p = 0.66

cohen.d(Coping$GAD.Total[Coping$Com_TV==1], Coping$GAD.Total[Coping$Com_TV==0], na.rm = TRUE) # d = 0.07

####Effective strategy & PHQ

#####Endorsers

mean(Coping$PHQ.Total[Coping$Eff_TV==1], na.rm = TRUE) # mean = 2.10

sd(Coping$PHQ.Total[Coping$Eff_TV==1], na.rm = TRUE) # SD = 2.28

#####Non-Endorsers

mean(Coping$PHQ.Total[Coping$Eff_TV==0], na.rm = TRUE) # mean = 2.03

sd(Coping$PHQ.Total[Coping$Eff_TV==0], na.rm = TRUE) # SD = 1.67

t.test(Coping$PHQ.Total[Coping$Eff_TV==1], Coping$PHQ.Total[Coping$Eff_TV==0], var.equal = TRUE) # p = 0.90

cohen.d(Coping$PHQ.Total[Coping$Eff_TV==1], Coping$PHQ.Total[Coping$Eff_TV==0], na.rm = TRUE) # d = -0.04

####Effective Strategy & GAD

#####Endorsers

mean(Coping$GAD.Total[Coping$Eff_TV==1], na.rm = TRUE) # mean = 2.60

sd(Coping$GAD.Total[Coping$Eff_TV==1], na.rm = TRUE) # SD = 2.17

#####Non-Endorsers

mean(Coping$GAD.Total[Coping$Eff_TV==0], na.rm = TRUE) # mean = 2.68

sd(Coping$GAD.Total[Coping$Eff_TV==0], na.rm = TRUE) # SD = 1.86

t.test(Coping$GAD.Total[Coping$Eff_TV==1], Coping$GAD.Total[Coping$Eff_TV==0], var.equal = TRUE) # p = 0.89

cohen.d(Coping$GAD.Total[Coping$Eff_TV==1], Coping$GAD.Total[Coping$Eff_TV==0], na.rm = TRUE) # d = 0.045

###Physical Activity

####Checking variance to ensure meets assumptions for t-tests

var.test(Coping$PHQ.Total[Coping$Com_Physical.Activity==1], Coping$PHQ.Total[Coping$Com_Physical.Activity==0]) #unequal variances

var.test(Coping$GAD.Total[Coping$Com_Physical.Activity==1], Coping$GAD.Total[Coping$Com_Physical.Activity==0])

var.test(Coping$PHQ.Total[Coping$Eff_Physical.Activity==1], Coping$PHQ.Total[Coping$Eff_Physical.Activity==0])

var.test(Coping$GAD.Total[Coping$Eff_Physical.Activity==1], Coping$GAD.Total[Coping$Eff_Physical.Activity==0])

####Common strategy & PHQ

#####Endorsers

mean(Coping$PHQ.Total[Coping$Com_Physical.Activity==1], na.rm = TRUE) # mean = 1.19

sd(Coping$PHQ.Total[Coping$Com_Physical.Activity==1], na.rm = TRUE) # SD = 1.19

#####Non-Endorsers

mean(Coping$PHQ.Total[Coping$Com_Physical.Activity==0], na.rm = TRUE) # mean = 2.23

sd(Coping$PHQ.Total[Coping$Com_Physical.Activity==0], na.rm = TRUE) # SD = 1.73

t.test(Coping$PHQ.Total[Coping$Com_Physical.Activity==1], Coping$PHQ.Total[Coping$Com_Physical.Activity==0]) # p = 0.0000003

cohen.d(Coping$PHQ.Total[Coping$Com_Physical.Activity==1], Coping$PHQ.Total[Coping$Com_Physical.Activity==0], na.rm = TRUE) # d = 0.63

####Common strategy & GAD

#####Endorsers

mean(Coping$GAD.Total[Coping$Com_Physical.Activity==1], na.rm = TRUE) # mean = 2.09

sd(Coping$GAD.Total[Coping$Com_Physical.Activity==1], na.rm = TRUE) # SD = 1.71

#####Non-Endorsers

mean(Coping$GAD.Total[Coping$Com_Physical.Activity==0], na.rm = TRUE) # mean = 2.82

sd(Coping$GAD.Total[Coping$Com_Physical.Activity==0], na.rm = TRUE) # SD = 1.88

t.test(Coping$GAD.Total[Coping$Com_Physical.Activity==1], Coping$GAD.Total[Coping$Com_Physical.Activity==0], var.equal = TRUE) # p = 0.008

cohen.d(Coping$GAD.Total[Coping$Com_Physical.Activity==1], Coping$GAD.Total[Coping$Com_Physical.Activity==0], na.rm = TRUE) # d = 0.39

####Effective strategy & PHQ

#####Endorsers

mean(Coping$PHQ.Total[Coping$Eff_Physical.Activity==1], na.rm = TRUE) # mean = 2.08

sd(Coping$PHQ.Total[Coping$Eff_Physical.Activity==1], na.rm = TRUE) # SD = 1.64

#####Non-Endorsers

mean(Coping$PHQ.Total[Coping$Eff_Physical.Activity==0], na.rm = TRUE) # mean = 2.01

sd(Coping$PHQ.Total[Coping$Eff_Physical.Activity==0], na.rm = TRUE) # SD = 1.73

t.test(Coping$PHQ.Total[Coping$Eff_Physical.Activity==1], Coping$PHQ.Total[Coping$Eff_Physical.Activity==0], var.equal = TRUE) # p = 0.70

cohen.d(Coping$PHQ.Total[Coping$Eff_Physical.Activity==1], Coping$PHQ.Total[Coping$Eff_Physical.Activity==0], na.rm = TRUE) # d = -0.05

####Effective Strategy & GAD

#####Endorsers

mean(Coping$GAD.Total[Coping$Eff_Physical.Activity==1], na.rm = TRUE) # mean = 2.88

sd(Coping$GAD.Total[Coping$Eff_Physical.Activity==1], na.rm = TRUE) # SD = 1.96

#####Non-Endorsers

mean(Coping$GAD.Total[Coping$Eff_Physical.Activity==0], na.rm = TRUE) # mean = 2.55

sd(Coping$GAD.Total[Coping$Eff_Physical.Activity==0], na.rm = TRUE) # SD = 1.80

t.test(Coping$GAD.Total[Coping$Eff_Physical.Activity==1], Coping$GAD.Total[Coping$Eff_Physical.Activity==0], var.equal = TRUE) # p = 0.14

cohen.d(Coping$GAD.Total[Coping$Eff_Physical.Activity==1], Coping$GAD.Total[Coping$Eff_Physical.Activity==0], na.rm = TRUE) # d = -0.17

##Associations between Top Problems and Mental Health

###checking variance to ensure meets assumptions for t-tests

var.test(Coping$PHQ.Total[Coping$PRODUCTIVITY...WORK==1], Coping$PHQ.Total[Coping$PRODUCTIVITY...WORK==0])

var.test(Coping$GAD.Total[Coping$PRODUCTIVITY...WORK==1], Coping$GAD.Total[Coping$PRODUCTIVITY...WORK==0])

var.test(Coping$PHQ.Total[Coping$HEALTH==1], Coping$PHQ.Total[Coping$HEALTH==0])

var.test(Coping$GAD.Total[Coping$HEALTH==1], Coping$GAD.Total[Coping$HEALTH==0])

t.test(Coping$PHQ.Total[Coping$PRODUCTIVITY...WORK==1], Coping$PHQ.Total[Coping$PRODUCTIVITY...WORK==0],

var.equal = TRUE) # p = 0.88

t.test(Coping$GAD.Total[Coping$PRODUCTIVITY...WORK==1], Coping$GAD.Total[Coping$PRODUCTIVITY...WORK==0],

var.equal = TRUE) # p = 0.07
